# Electromagnetic scattering from random rough surface using higher-order GTD-RT numerical technique for optical wireless communications

**DOI:** 10.1038/s41598-025-07694-z

**Published:** 2025-07-02

**Authors:** Asmaa E. Farahat, Khalid F. A. Hussein

**Affiliations:** Microwave Engineering Department, Electronics Research Institute, Cairo, 11843 Egypt

**Keywords:** Electrical and electronic engineering, Computational science

## Abstract

This study introduces new approach which combines the geometrical theory of diffraction (GTD) and the ray tracing (RT) method to analyze the produced scattering pattern due to a striking plane wave on a rough surface with random attributes regarding electromagnetic and statistical properties. The Fresnel equation-based model is utilized to determine the distribution of scattered power for both reflection from the region above the surface and transmission into the region beneath the surface. The polarization (direction of electric field) of the incident optical wave is also considered. The proposed algorithm addresses multi-bounce of striking ray, making it an advanced higher-order GTD-RT approach. The precision of the findings is validated by comparing them with scattering pattern data from empirical observations produced by a Radiant beam striking paper sheets with different roughness properties and different probabilistic characteristics. This study’s numerical results explore how the scattering pattern is influenced by surface degree of granularity, incidence angle, and light refraction coefficient of the rough surface. Additionally, first and second order scattering are calculated and compared. The second-order GTD-RT method provides slightly improved accuracy over the first-order method, especially for highly rough surfaces, but the enhancement remains marginal. Given its low average error (< 2.5%) and significantly lower computational cost, the first-order GTD-RT method offers a more practical and efficient solution for optical scattering analysis in rough surface scenarios. To validate the accuracy and reliability of the proposed model, a subset of the numerical results obtained in this study has been systematically compared with previously published findings derived using alternative analytical approaches, specifically the Geometrical Optics (GO) method and the second-order Kirchhoff approximation. These comparisons serve to highlight the consistency of the presented approach with established theoretical models and underscore its capability to accurately characterize the scattering behavior from rough surfaces under similar conditions.

## Introduction

Optical Wireless Communication (OWC) uses optical waves, including infrared, visible, and ultraviolet spectra, to transmit data through free space. This technology offers high data rates and immunity to electromagnetic interference, making it a promising solution for various applications, from indoor networking to underwater communication. However, the performance of OWC systems is heavily influenced by environmental factors, particularly the interaction of light with surfaces in the propagation path. Understanding how rough surfaces scatter light is crucial for optimizing OWC system design and ensuring reliable data transmission. When light encounters a rough surface, it undergoes scattering, redistributing the light energy in multiple directions. This scattering can degrade signal strength and quality, especially in non-line-of-sight (NLOS) communication scenarios where the direct path between transmitter and receiver is obstructed. For instance, in ultraviolet (UV) communication systems, ground surfaces with varying roughness can significantly affect the amount of scattered light reaching the receiver. Studies have shown that as the elevation angle of the transmitter decreases, more UV photons are scattered by the ground, contributing to the received signal. Accurately modeling this scattering behavior is essential for predicting path loss and optimizing system performance^1,2^.

In indoor OWC systems, rough surfaces like walls and furniture cause diffuse scattering, leading to multipath effects and signal fading. Accurate channel models that consider surface roughness are essential to improve communication reliability. Advanced materials, such as reconfigurable intelligent surfaces (RIS), can control light scattering and enhance signal strength and coverage by dynamically adjusting surface properties, effectively mitigating the impact of obstacles and rough surfaces^2,3^. A thorough understanding of scattering phenomena enables the development of accurate channel models, effective mitigation strategies, and innovative technologies, all of which contribute to the reliability and efficiency of OWC systems across various applications.

Recent advancements in optical wireless communication (OWC) have utilized a range of analytical and semi-analytical models to better understand and mitigate the impact of scattering caused by rough surfaces. Beyond traditional OWC research, developments in other areas of optical communication offer valuable insights that can inform future strategies. For instance, the work presented in^4^ introduces an innovative end-to-end learning approach for optimizing hollow-core fiber transmission, which could inspire data-driven methods for improving OWC systems under multipath and scattering effects. Additionally, the study in^5^ explores in-band noise modulation to enhance signal-to-noise ratio (SNR), suggesting that similar modulation techniques might be adapted to reduce the adverse effects of surface-induced interference in OWC environments. Incorporating such interdisciplinary concepts can expand the theoretical foundation and open new directions for optimizing OWC system performance.

Optical wireless communication (OWC) systems have introduced a variety of sophisticated modulation and anti-interference techniques to enhance transmission efficiency and reliability. Techniques such as low-PAPR layered/enhanced ACO-SCFDM have been proposed to reduce signal distortion and improve power efficiency^6^. Orthogonal time–frequency multiplexing with 2D Hermitian symmetry offers increased spectral efficiency and robustness against interference^7^. Additionally, the integration of VCSEL-based devices has enabled high-speed, multi-user OWC links, demonstrating practical feasibility for dense communication environments^8^.

Analytical and semi-analytical methods, including small-amplitude perturbation theory, Kirchhoff approximation, extinction theorem, and Rayleigh scattering techniques, are used to study electromagnetic scattering from rough surfaces^9–16^. The Beckmann–Kirchhoff theory links the scattered light pattern to surface statistical features, enabling the estimation of the height autocorrelation function^14^. The extinction theorem calculates angular distributions of scattered fields using Monte Carlo simulations for rough surface profiles^15^. Additionally, the reduced Rayleigh equation models light scattering from 2D porous rough surfaces, considering different polarization states and surface statistical properties^16^. An overview of additional methods for evaluating electromagnetic diffraction from irregular surfaces is presented in^17^. The techniques discussed include the full-wave approach, phase-perturbation technique, Wiener–Hermite process, enhanced Green’s function techniques, and the integral equation method, and others.

This paper utilizes the Ray Tracing (RT) merged with Geometrical Diffraction Theory (GTD) to model the interaction of electromagnetic/optical waves with irregular surfaces. This GTD-RT method takes into account higher-order scattering through successive reflections of rays incident on the rough surface. It also calculates Fresnel reflection and transmission coefficients to incorporate the impact of the irregular surface material, making it used for random rough surfaces, regardless of being metallic or dielectric types with arbitrary probabilistic properties. Unlike analytical or semi-analytical methods, this fully numerical approach avoids approximations that can lead to inaccuracies, except for the high-frequency assumptions inherent to ray theory. The scattering patterns obtained by the GTD-RT technique are validated using practical measurements. The model also considers the polarization of the incident plane wave. Additionally, first and second order scattering are calculated and compared. It is found that the numerical error of accounting for the higher order bounces increases by increasing the surface roughness.

## Rough surface modeling

To numerically evaluate a single-diffuse indoor WOC system, a geometric model is needed to simulate a matte paint layer on the room’s interior walls. Analyzing optical wave scattering from these diffuse surfaces necessitates creating a random rough surface model with isotropic statistical characteristics. This section outlines a straightforward spatial-domain technique for generating a random rough surface with specified statistical properties.

Gaussian rough surfaces, characterized by a normal distribution of surface heights and a Gaussian correlation function, are considered single-scale surfaces due to their band-limited spectrum. Their well-defined statistical properties have made them a popular choice for extensive research in rough surface scattering studies. These surfaces are fully described by two statistical parameters: ${L}_{c}$ and ${h}_{rms}$, where ${L}_{c}$ is the surface length of correlation between adjacent points and ${h}_{rms}$ is the surface root mean square latitude. For this analysis, it is considered that the rough surfaces are isotropic, a power exponential or Gaussian shape is assigned to the correlation function, as given below.

1 $$C\left( d \right) = h_{rms}^{2} {\text{exp}}\left( { - \frac{{d^{2} }}{{L_{c}^{2} }}} \right){ },$$

where $d$ represents the horizontal separation between two points that are correlated on the surface.

The surface steepness could be determined using the second-order moment of the power spectrum. In the context of a rough surface with a Gaussian correlation model, the slope is given by $s=\sqrt{2}{h}_{rms}/{L}_{c}$. In this work, the surface roughness is defined as follows.

2 $$R_{D} = \sqrt 2 s = \frac{{2h_{rms} }}{{L_{c} }}$$

We believe that the parameter ${R}_{D}$ is a more suitable representation of surface roughness.

Consider a surface with dimensions ${L}_{x}\times {L}_{y}$, discretized into ${Q}_{x}\times {Q}_{y}$​ points along $x$ and $y$ axes. The correlation lengths in these directions are ${L}_{cx}$​ and ${L}_{cy}$, respectively. Each surface point is defined by coordinates $\left(x,y, z\right)$, where $z$ represents the height with random variation, and $x$ and $y$ form a grid evenly spaced in the horizontal plane. The horizontal spacing between neighboring points is $\Delta x$ and $\Delta y$ along the $x$ and $y$ directions. The surface roughness is influenced by the ratio of ${h}_{rms}$​ to the correlation length. In the case of a square surface with isotropic properties, where ${L}_{cx}={L}_{cy}={L}_{c}$, ${L}_{x}={L}_{y}=L$, ${Q}_{x}={Q}_{y}=Q$, and $\Delta x=\Delta y=\Delta$, the number of points per correlation length is denoted as ${N}_{Lc}$ ​. Consequently, the correlation length ${L}_{c}$ is evaluated accordingly.

3 $$L_{c} = \left( {N_{Lc} - 1} \right){ }\Delta$$

To numerically create rough surface, a 2D matrix of random values following a Gaussian distribution with a mean of zero,$\mu =0$, is created and a standard deviation of $={h}_{rms}$​. These values correspond to the elevations at specific locations on the surface and are initially uncorrelated. To achieve the desired correlation length, a Savitzky-Golay filter (SGF) is employed with a defined range of influence. This filter, characterized by a size denoted as ${N}_{Lc}$​, is systematically applied in a sequential manner. Initially, it is implemented across the rows of the array, ensuring that the smoothing effect is introduced along one dimension. Following this, the same filtering process is applied to the columns, further refining the data structure and maintaining the intended correlation characteristics^18^. The mean value of the smoothed array is then subtracted from each element, and the values are scaled to achieve the desired root-mean-square height. It has been demonstrated using variogram analysis that this method accurately produces a rough surface model with the specified statistical parameters (${L}_{c}$ and ${h}_{rms}$​) and a Gaussian correlation function^18^.

## Scattering of optical waves incident on random rough surfaces

**Fig. 1:**
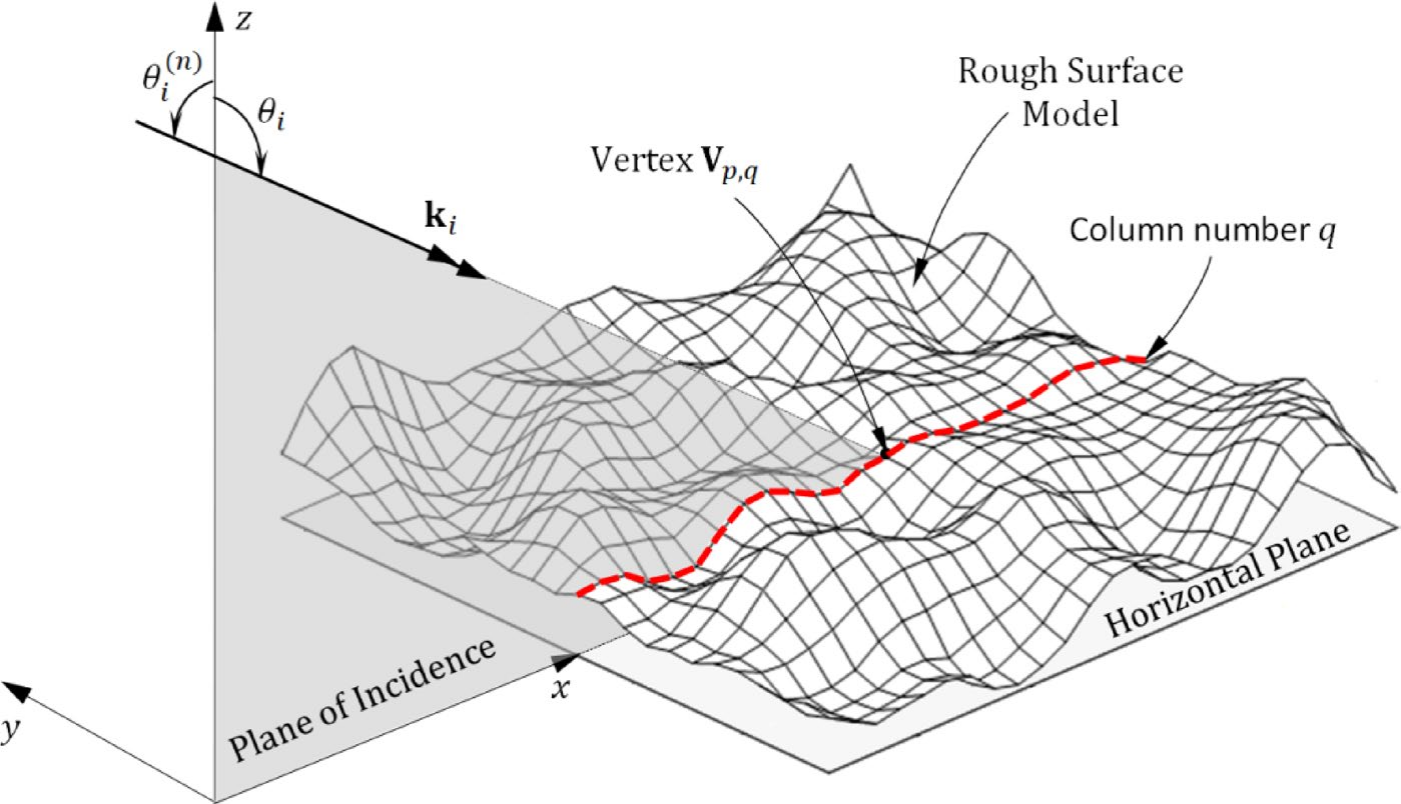
Every vertex in the column number $q$ of the surface representation lies on the same plane and reside within the incidence plane, defined by $y=\left(q-1\right) \Delta y$.

This section is dedicated to the analysis of optical wave scattering caused by random rough surfaces that exhibit arbitrary statistical characteristics. The study employs a combination of the Geometrical Theory of Diffraction (GTD) and the Ray Tracing (RT) method to accurately model and evaluate the scattering phenomena. By integrating these approaches, the investigation aims to provide a comprehensive understanding of how optical waves interact with complex surface geometries, capturing both the direct and diffracted contributions to the overall scattering behavior. The structural representation of the rough surface follows the methodology described in "Rough surface modeling" section, where the incidence plane is positioned aligned with the x–z plane, as shown in Fig. 1. An incoming plane wave is classified based on the orientation of its electric field. It is termed vertically polarized (V-polarized) when the electric field is confined within the incidence plane. On the other hand, if the electric field is entirely contained within a plane parallel to the x–y plane, the wave is referred to as horizontally polarized (H-polarized). The incoming wave’s approach is determined by the angle ${\theta }_{i}$​, which specifies the relationship between the propagation vector kik_iki​ and the positive z-axis. Additionally, the conventional incidence angle, represented as ${\theta }_{i}^{(n)}$​, is defined as the supplementary counterpart of ${\theta }_{i}$​. This differentiation aids in understanding wave interactions with irregular surfaces under various angular conditions.

### GTD-RT method suitability for calculating scattering of optical waves

For the approximate technique of geometric optics underlying the approach of GTD to provide accurate electromagnetic scattering predictions, the wavelength must be significantly smaller than the characteristic features of the scattering surface, including irregularities and curves. Therefore, when analyzing scattering from a rough surface using the GTD-RT method, it is essential to ensure that specific conditions are met for reliable results.

4a $$\lambda < < L_{c} \sqrt {1 + R_{D}^{2} }$$

The last condition (4a) is likely satisfied when $\lambda \sim 10 {L}_{c}\sqrt{1+{R}_{D}^{2}}$. However, practical work in "Results and discussions" section of this study demonstrate that reliable outcomes can still be obtained by GTD-RT method for $\lambda \sim {L}_{c}\sqrt{1+{R}_{D}^{2}}$​ such as when $\lambda =2 {L}_{c}$. Additionally, it is noted in^19^ that the geometric optics (GO) approximation can provide reliable results for rough surface scattering under certain conditions, even when $\lambda > {L}_{c}\sqrt{1+{R}_{D}^{2}}$, for example, when $\lambda \approx 0.65 {L}_{c}$)​.​ For precise numerical evaluation of scattering of optical rays by the GTD-RT method, this requirement must be met.

4b $$\lambda > > \Delta$$

When the conditions specified in (4) are met, the GTD-RT method becomes applicable for examining the interaction between an optical ray or a collection of rays and the rough surface representation, allowing the scattering to be determined.

### Utilizing GTD-RT for evaluating scattering of plane waves

The analysis in this part of the paper explains the proposed GTD-RT method for analyzing optical and electromagnetic scattering from an incident plane wave, as illustrated in Fig. 1. Multiple rays representing the plane wave are assumed incident on the rough surface, each carrying a portion of power to ensure a balanced power spreading. The phase of a scattered ray is calculated based on the overall length of the distance traveled throughout ray tracing. The surface’s reflective and absorptive characteristics are determined using Fresnel coefficients at the point of wave striking the surface. Additionally, a procedure is introduced to compute the distribution of scattered waves in the upper region. The approach includes ray tracing of both primary and advanced orders, presented by an algorithm suitable for simulating optical and electromagnetic wave scattering.

The surface is represented as a network of evenly distributed points in the *x* and *y* directions, with intervals of $\Delta x$ and $\Delta y$, respectively. There are $P$ vertices along the *x* -axis and $Q$ along the *y*-axis. Every point in this grid is assigned an unpredictable elevation, ${z}_{p,q}$, where $p$ and $q$ denote the row and column indices in the $x-$ and $y-$ directions, respectively. Therefore, the location of a point on the surface can be represented as a position vector:

5 $${\mathbf{V}}_{p,q} = x_{p,q} {\hat{\mathbf{a}}}_{x} + y_{p,q} {\hat{\mathbf{a}}}_{y} + z_{p,q} {\hat{\mathbf{a}}}_{z}$$

6 $$x_{p,q} = p\Delta x , y_{p,q} = q\Delta y$$

In the grid expressing the surface, each vertex is linked to its neighboring vertices through triangular meshing. The perpendicular vector at each point is obtained by averaging the normals of the surrounding triangular facets that include that point. The steps for implementing the proposed technique are outlined in the subsequent sections.

#### Modeling of the incoming plane wave with incident rays

The incident plane wave’s propagation constant can be represented as,

7 $${\mathbf{k}}_{i} = k_{o} {\mathbf{\hat{a}}}_{{k_{i} }} = k_{o} \left( {{\mathbf{\hat{a}}}_{x} k_{{i_{x} }} + {\mathbf{\hat{a}}}_{z} k_{{i_{z} }} } \right),$$

Here, ${k}_{o}$​ denotes the free space wavenumber, and ${\widehat{\mathbf{a}}}_{{k}_{i}}$​​ is the normalized vector indicating the direction of incidence.

8 $$k_{{i_{x} }} = \sin \theta _{i} ,{\text{~~}}k_{{i_{z} }} = \cos \theta _{i}$$

The striking wave is modeled using series of parallel rays, with each ray stiking on a vertex ${\mathbf{V}}_{p,q}$ of the surface. Each ray carries a power ${{\mathcalligra{p}}_{i}}_{p,q}$​​ calculated to ensure a uniform power density distribution across the transverse plane. For simplicity, The plane wave is considered to have a normalized direction vector for propagation, ${\hat{\mathbf{a}}}_{{k_{i} }}$, lies within the $x$*-*$z$ plane. Let the transverse plane, which is perpendicular to ${\widehat{\mathbf{a}}}_{{k}_{i}}$ and goes through the point ${\mathbf{O}}_{q}=({0,y}_{p,q},0)$, intersects the plane $y={y}_{p,q}$ along the line ${{\mathcalligra{L}}_{i}}_{q}$ ​​, which also passes through the point ${\mathbf{O}}_{q}$​.

#### Determining the power linked to each incoming ray

**Fig. 2:**
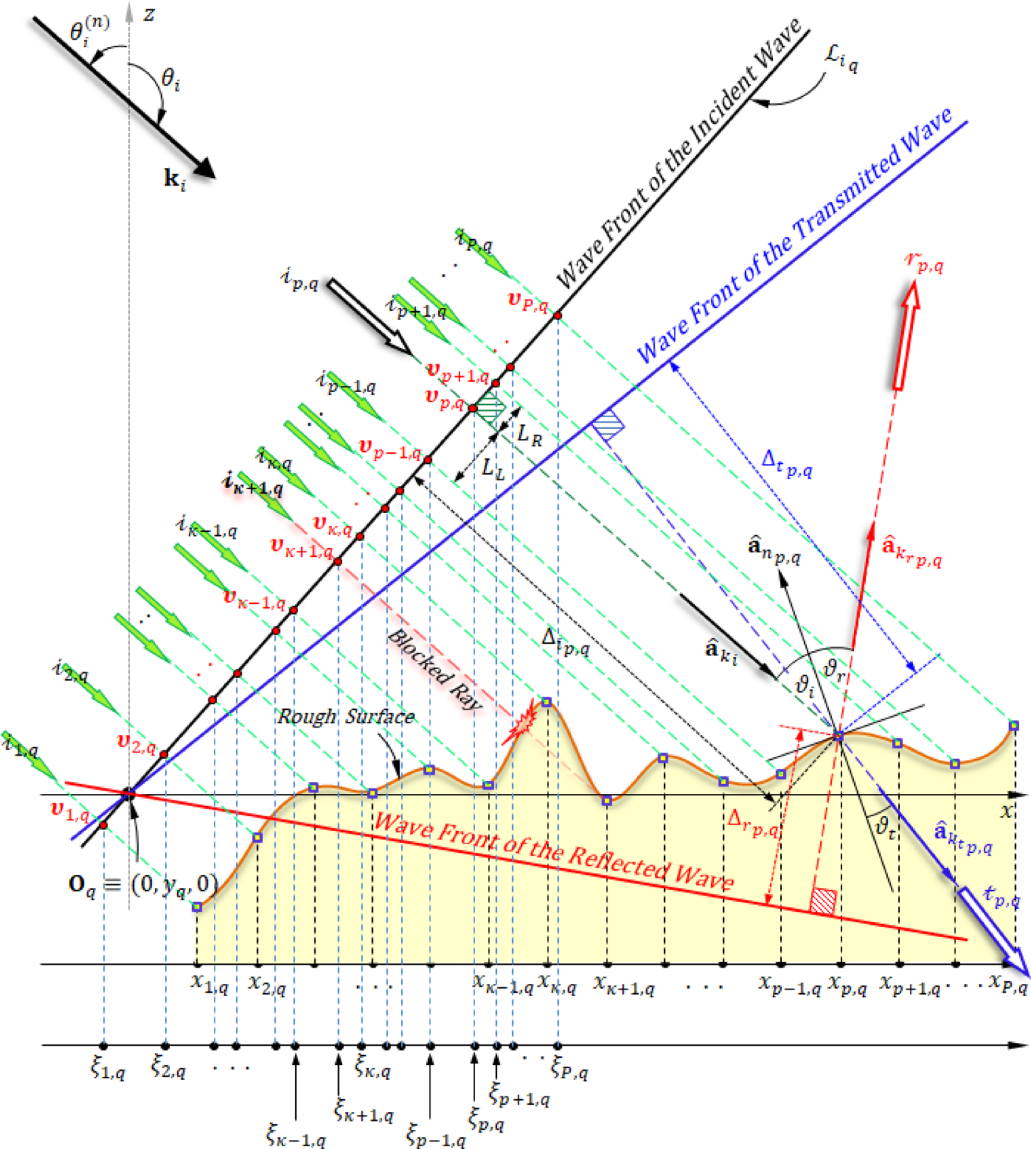
The ray tracing approach utilized to evaluate how plane waves scatter when they encounter rough surfaces, $y=\left(q-1\right) \Delta y$ is the incidence plane.

Beginning with the first line of points (vertices) on the grid that models the surface and progressing row by row to the ${Q}^{\text{th}}$ row, the points on the ${q}^{\text{th}}$ row, ${\mathbf{V}}_{p,q}$, are scanned in order from $p=1$ to $p=P$ using the following procedure:

1. From each point representing a vertex ${\mathbf{V}}_{p,q}$, Construct a line that is orthogonal to the incident wave’s transverse plane, as illustrated in Fig. 2. Get the intersection vertex ${{\varvec{\upupsilon}}}_{p}$​, with its position vector defined relative to the point ${\mathbf{O}}_{q}$​ is given by ${{\varvec{\upupsilon}}}_{p}=({\xi }_{p,q}, 0,{ \zeta }_{p,q})$. This can be calculated as follows:
  9 $${\mathbf{\upsilon }}_{p} \cdot {\mathbf{\hat{a}}}_{{k_{i} }} = 0 = \xi _{{p,q}} ~k_{{i_{x} }} + {\text{~}}\zeta _{{p,q}} ~k_{{i_{z} }}$$
  Let ${\Delta }_{i}$ denote the separation between the intersection point ${{\varvec{\upupsilon}}}_{p}$ and the vertex ${\mathbf{V}}_{p,q}$*.* Therefore, it can be expressed as:
  10 $${\mathbf{V}}_{p,q} = {{\varvec{\upupsilon}}}_{p} + {{ \Delta }}_{{i_{p,q} }} { }{\hat{\mathbf{a}}}_{{k_{i} }}$$
  This equation can be broken down into the following scalar components:
  11a $$\xi _{{p,q}} = x_{{p,q}} - k_{{i_{x} {\text{~}}}} {{\Delta }}_{{i_{{p,q}} }}$$
  11b $$\zeta _{{p,q}} = z_{{p,q}} - k_{{i_{z} }} {{\Delta }}_{{i_{{p,q}} }}$$
  Equations (9), (11a), and (11b) can be solved simultaneously to find the values of ${{\Delta }_{i}}_{p,q}$, ${\xi }_{p,q}$ and ${\zeta }_{p,q}$ as follows. By substituting (11a) and (11b) into (9) and utilizing (8), we obtain:
  12 $$\Delta_{{i_{p,q} }} = x_{p,q} \sin \theta_{i} + z_{p,q} \cos \theta_{i}$$
2. Set ${\xi }_{max}$ ​ as the highest *x*-component value of ${{\varvec{\upupsilon}}}_{p}$ encountered while sequentially examining the vertices on the ${q}^{\text{th}}$ row from $p=1$ to $p=P$. Start with ${\xi }_{max}=0$. Additionally, introduce a flag variable ${\Upsilon }_{p,q}$​ to indicate the status of the incident ray at the corresponding vertex.
3. As the points on the ${q}^{\text{th}}$ line are scanned sequentially moving from the left side to right side, $p$ increments by one for each point, and the crossing point ${{\varvec{\upupsilon}}}_{p}$ is determined as previously explained. ${\xi }_{max}$ then takes ne value accordingly. Specifically, if ${\xi }_{p,q}>{\xi }_{max}$​, the ray is marked as active (${\Upsilon }_{p,q}=1$), and ${\xi }_{max}$ is updated to ${\xi }_{max}={\xi }_{p,q}$. Conversely, if ${\xi }_{p,q}\le {\xi }_{max}$, the ray is deemed inactive (${\Upsilon }_{p,q}=0$), indicating that the point ($p,q$) is blocked from the incoming wave by another portion of the surface. As a result, the ray ${i}_{p,q}$​ has no contribution in the scattered field calculation, and its associated power is considered zero. Consequently, as illustrated in Fig. 2, the illuminated area corresponding to each coming ray striking the transverse plane varies and calculated as follows.
  13 $${\Lambda }_{p,q} = \frac{1}{2}{ }\left( {L_{R} + L_{L} } \right){{ \Delta }}y{ }$$

Here, ${L}_{R}$​ represents the separation among the vertex ${{\varvec{\upupsilon}}}_{p}$ and the closest vertex to its right, ${{{\varvec{\upupsilon}}}_{p}}_{R}$, on ${\mathcalligra{L}}_{{i_{q} }}$ associated with the engaged ray. Similarly, ${L}_{L}$​ denotes the separation among ${{\varvec{\upupsilon}}}_{p}$​ and the nearest vertex to its left, ${{{\varvec{\upupsilon}}}_{p}}_{L}$, also associated with an active ray. Therefore, the following relationship holds:

14a $$L_{R} = \left| {{\varvec{\upsilon }}_{{p_{R} }} - {\varvec{\upsilon }}_{p} } \right|$$

14b $$L_{L} = \left| {{{\varvec{\upupsilon}}}_{{p_{L} }} - {{\varvec{\upupsilon}}}_{p} } \right|$$

To maintain even power distribution on the transverse plane, the power linked to each ray, ${{\mathcalligra{p}}_{i}}_{p,q}$, must be adjusted in relation to ${\Lambda }_{p,q}$​. Thus, it can be written as:

15 $$\mathcalligra{p}_{{i_{p,q} }} = {\Lambda }_{p,q} { }P_{d} { }$$

Here, ${P}_{d}$ denotes the power density of the incoming incident striking wave.

#### Directions and phases of reflected rays

In analyzing scattering at a microscopic level, the area around the point of incidence is considered to be nearly flat, enabling the use of Snell’s law to determine the reflected ray’s orientation. When an incident ray ${\mathcalligra{i}}_{p,q}$ strikes the surface at vertex ${\mathbf{V}}_{p,q}$, it forms an angle ${\vartheta }_{i}$​ with the normal vector ${\hat{\mathbf{a}}}_{{n_{p,q} }}$​ at that point. The corresponding reflected ray, ${\mathcalligra{r}}_{p,q}$, remains in the same plane as the incident direction and the normal vector, preserving the angle ${\vartheta }_{r}={\vartheta }_{i}$​, as illustrated in Fig. 1.

16 $${\hat{\mathbf{a}}}_{{k_{{r_{p,q} }} }} = {\hat{\mathbf{a}}}_{{k_{i} }} + 2\cos \vartheta_{i} {\hat{\mathbf{a}}}_{{n_{p,q} }}$$

The unit vector for the reflected wave, denoted as ${\hat{\mathbf{a}}}_{{k_{{r_{p,q} }} }}$ can be expressed through its individual components as follows:

17 $${\hat{\mathbf{a}}}_{{k_{{r_{p,q} }} }} = k_{{rx_{p,q} }} {\hat{\mathbf{a}}}_{x} + k_{{ry_{p,q} }} {\hat{\mathbf{a}}}_{y} + k_{{rz_{p,q} }} {\hat{\mathbf{a}}}_{z}$$

To determine the associated phase linked to each back ray ${\mathcalligra{r}}_{p,q}$ reflected from the surface, it is necessary to calculate the path difference, denoted as ${{ \Delta }}_{{r_{p,q} }}$, as illustrated in Fig. 2. From each vertex ${\mathbf{V}}_{p,q}$​, a line perpendicular to the wavefront of the back wave is sketched, as depicted in Fig. 2. The task is to find the crossing location of ${{\varvec{\upnu}}}_{p}$ with respect to the point ${\mathbf{O}}_{q}$​ is given by ${{\varvec{\upnu}}}_{p,q} = \left( {\alpha_{p,q} ,{ }\beta_{p,q} ,{ }\gamma_{p,q} } \right)$. This can be accomplished using the following approach.

18 $${{\varvec{\upnu}}}_{p,q} \cdot {\hat{\mathbf{a}}}_{{k_{{r_{p,q} }} }} = 0 = \alpha_{p,q} k_{{rx_{p,q} }} + { }\beta_{p,q} k_{{ry_{p,q} }} + { }\gamma_{p,q} k_{{rz_{p,q} }}$$

Define ${{ \Delta }}_{{r_{p,q} }}$ as the separation between ${\mathbf{V}}_{p,q}$ and ${{\varvec{\upnu}}}_{p,q}$. Consequently, the following expression is obtained:

19 $${\mathbf{\nu }}_{{p,q}} = {\mathbf{V}}_{{p,q}} - {{\Delta }}_{{r_{{p,q}} }} {\mathbf{\hat{a}}}_{{k_{{r_{{p,q}} }} }}$$

This can be expressed as the following scalar equations:

20a $$\alpha _{{p,q}} = x_{{p,q}} - ~k_{{rx_{{p,q}} }} {{\Delta }}_{{r_{{p,q}} }}$$

20b $$~\beta _{{p,q}} = y_{{p,q}} - ~k_{{ry_{{p,q}} }} ~\Delta _{{r_{{p,q}} }}$$

20c $$~\gamma _{{p,q}} = z_{{p,q}} - ~k_{{rz_{{p,q}} }} ~\Delta _{{r_{{p,q}} }}$$

By solving Eqs. (18), (20a), (20b), and (20c) simultaneously, the value of ${{\Delta }_{r}}_{p,q}$, can be obtained as:

21 $${{ \Delta }}_{{r_{p,q} }} { } = x_{p,q} k_{{rx_{p,q} }} + x_{p,q} k_{{ry_{p,q} }} + z_{p,q} k_{{rz_{p,q} }}$$

Therefore, the phase corresponding to ${\mathcalligra{r}}_{p,q}$ is given by:

22 $${{\Delta \Phi }}_{{r_{p,q} }} = \frac{2\pi }{\lambda }\left( {{{ \Delta }}_{{i_{p,q} }} - {{ \Delta }}_{{r_{p,q} }} } \right)$$

#### Directions and phases of transmitted rays

At the vertex ${\mathbf{V}}_{p,q}$, the transmitted ray ${\mathcalligra{t}}_{p,q}$ is positioned within the plane formed by the incident wave vector ${\widehat{\mathbf{a}}}_{{k}_{i}}$ and the surface normal ${\widehat{\mathbf{a}}}_{{n}_{p,q}}$. It makes an angle ${\vartheta }_{t}$ with the inward-pointing normal vector $-{\widehat{\mathbf{a}}}_{{n}_{p,q}}$ according to Snell’s law. The connection between ${\vartheta }_{i}$ and ${\vartheta }_{t}$ is represented by:

23 $$n_{1} \sin \vartheta_{i} = n_{2} \sin \vartheta_{t}$$

Therefore, the direction of ray ${\mathcalligra{t}}_{p,q}$ is represented by the unit vector ${\hat{\mathbf{a}}}_{{k_{{t_{p,q} }} }}$ calculated as follows.

24 $${\hat{\mathbf{a}}}_{{k_{{t_{p,q} }} }} = \frac{{n_{1} }}{{n_{2} }} {\hat{\mathbf{a}}}_{{k_{i} }} + \left( {\frac{{n_{1} }}{{n_{2} }}\cos \vartheta_{i} - \cos \vartheta_{t} } \right){\hat{\mathbf{a}}}_{{n_{p,q} }}$$

The unit vector ${\hat{\mathbf{a}}}_{{k_{{t_{p,q} }} }}$ can be broken down into its individual components as shown below.

25 $${\mathbf{\hat{a}}}_{{k_{{t_{{p,q}} }} }} = ~k_{{tx_{{p,q}} }} {\mathbf{\hat{a}}}_{x} + ~k_{{ty_{{p,q}} }} ~{\mathbf{\hat{a}}}_{y} + ~k_{{tz_{{p,q}} }} ~{\mathbf{\hat{a}}}_{z}$$

The phase for each transmitted ray ${\mathcalligra{t}}_{p,q}$​ depends on its path difference ${{ \Delta }}_{{t_{p,q} }}$, as depicted in Fig. 2. By applying a method analogous to that used for calculating the path difference ${{ \Delta }}_{{r_{p,q} }}$, the following formula for ${{ \Delta }}_{{t_{p,q} }}$ is derived.

26 $${{ \Delta }}_{{t_{p,q} }} { } = x_{p,q} k_{{tx_{p,q} }} + x_{p,q} k_{{ty_{p,q} }} + z_{p,q} k_{{tz_{p,q} }}$$

Therefore, the phase of ${\mathcalligra{t}}_{p,q}$​ can be calculated using the following equation:

27 $$\Delta \Phi _{{t_{{p,q}} }} = \frac{{2\pi }}{\lambda }\left( {~\Delta _{{i_{{p,q}} }} - ~\Delta _{{t_{{p,q}} }} } \right)$$

#### Fresnel law at the interface

**Fig. 3:**
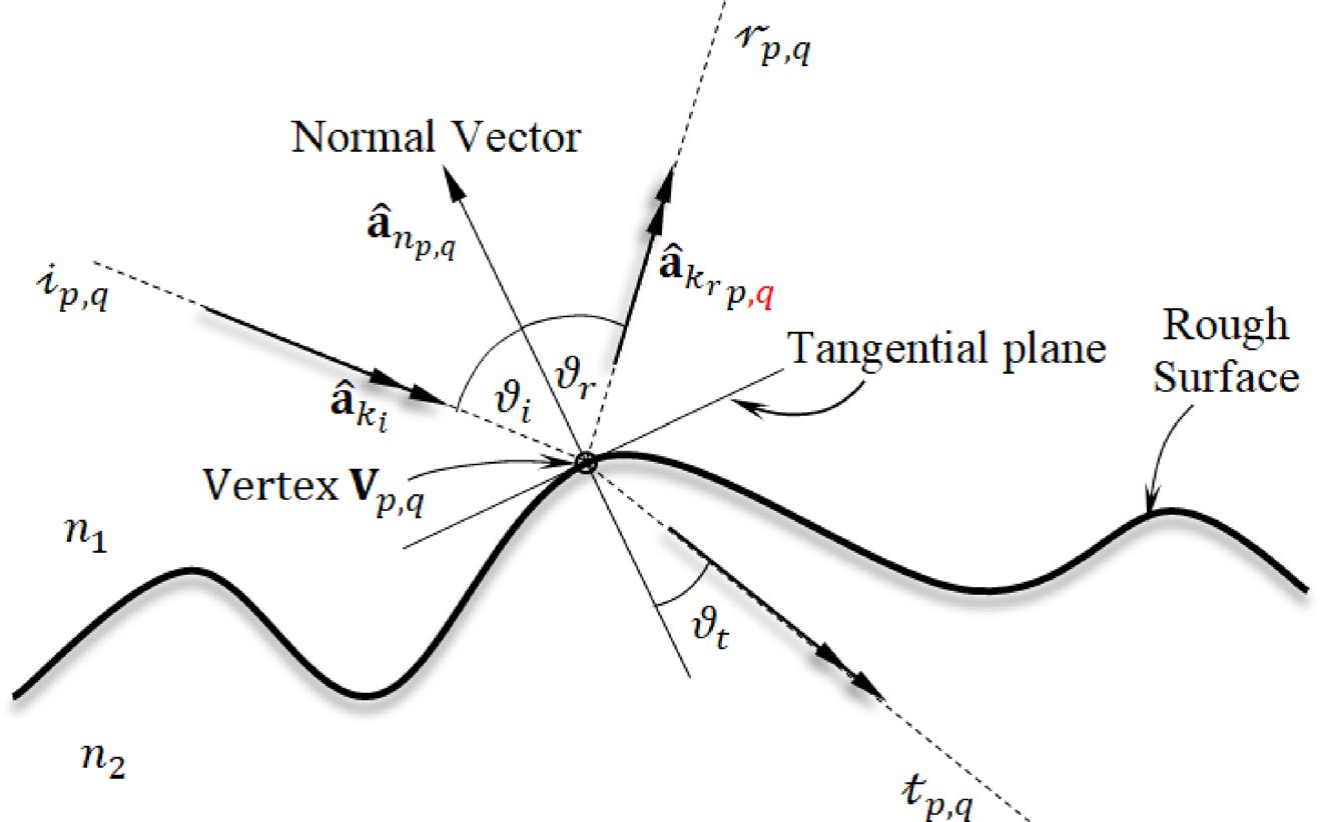
Fresnel law at an interface point.

At the point of impact ${\mathbf{V}}_{p,q}$, the incident ray ${\mathcalligra{i}}_{p,q}$, is both reflected and transmitted, as depicted in Fig. 3. The bouncing (reflected) and passing (transmitted) rays’ orientations depend on the surface’s normal at this location and the angles ${\vartheta }_{i}$, ${\vartheta }_{r}$, ${\vartheta }_{t}$​ illustrated in the figure. These angles are calculated using Snell’s law.

The power $\mathcalligra{p}_{{r_{p,q} }}$​ linked to the ray $\mathcalligra{r}_{p,q}$ at the point ${\mathbf{V}}_{p,q}$ has relation to the power of the incoming ray $\mathcalligra{i}_{p,q}$ at that point by the following equation.

28 $$\mathcalligra{p}_{{r_{p,q} }} = \left| {{\Gamma }_{p,q} } \right|^{2} _{{i_{p,q} }} ,$$

In first-order ray tracing, the term ${\left|{\Gamma }_{p,q}\right|}^{2}$ represents the reflectance, where ${\Gamma }_{p,q}$ is the Fresnel reflection coefficient at the vertex ${\mathbf{V}}_{p,q}$ for the incident ray ${\mathcalligra{i}}_{p,q}$. The definition of ${\Gamma }_{p,q}$ is expanded to a more comprehensive form at ray tracing of higher-order.

Now, the electric field vector hitting the surface can be separated into two components, ${E}_{h}^{i}$ and ${E}_{v}^{i}$, with respect to the surface at the location of impact, as shown below.

29 $${\mathbf{E}}^{i} = E_{h}^{i} {\hat{\mathbf{a}}}_{{hi_{p,q} }} E_{v}^{i} {\hat{\mathbf{a}}}_{{vi_{p,q} }} hi$$

Here, suffix ‘*v*’ denotes the vertical field component and suffix ‘*h*’ denote the horizontal field component, ${\hat{\mathbf{a}}}_{{hi_{p,q} }}$ and ${\hat{\mathbf{a}}}_{{vi_{p,q} }}$ represent the local unit vectors for horizontal and vertical polarization. These vectors are determined by.

30 $${\hat{\mathbf{a}}}_{{hi_{p,q} }} = \frac{{{\hat{\mathbf{a}}}_{{n_{p,q} }} \times { }{\hat{\mathbf{a}}}_{{k_{i} }} }}{{\sin \vartheta_{i} }}, {\hat{\mathbf{a}}}_{{vi_{p,q} }} = {\hat{\mathbf{a}}}_{{k_{i} }} \times {\hat{\mathbf{a}}}_{{hi_{p,q} }}$$

This involves breaking down the incoming electromagnetic field at each point on the surface into its individual elements, as described below.

31 $$E_{v}^{i} = {\mathbf{E}}^{i} \cdot {\hat{\mathbf{a}}}_{{vi_{p,q} }} ,\user2{ }E_{h}^{i} = {\mathbf{E}}^{i} \cdot {\hat{\mathbf{a}}}_{{hi_{p,q} }}$$

When the incoming electromagnetic field lies entirely within the incidence plane characterized by its vertical polarization, this plane is determined by the directional vectors ${\hat{\mathbf{a}}}_{{n_{p,q} }}$​​ and ${\hat{\mathbf{a}}}_{{k_{i} }}$​​. Under these conditions, the Fresnel coefficients for reflection and transmission, denoted as $\Gamma$ and $\text{T}$, respectively, can be mathematically represented using the equations.

32a $${\Gamma } = {\Gamma }_{v} = { }\frac{{E_{v}^{r} }}{{E_{v}^{i} }} = { }\frac{{n_{1} \cos \vartheta_{t} - n_{2} \cos \vartheta_{i} }}{{n_{1} \cos \vartheta_{t} + n_{2} \cos \vartheta_{i} }},$$

32b $${\text{T}} = {\text{T}}_{v} = { }\frac{{E_{v}^{t} }}{{E_{v}^{i} }} = { }\frac{{2n_{1} \cos \vartheta_{i} }}{{n_{1} \cos \vartheta_{t} + n_{2} \cos \vartheta_{i} }},$$

In these formulas, ${n}_{1}$ and ${n}_{2}$​ denote the optical density parameters (refractive indices) of the surrounding materials on either side of the surface. If the electric field of the incoming wave is oriented perpendicular to the incidence plane (horizontal polarization), the Fresnel coefficients are defined as

33a $${\Gamma } = {\Gamma }_{h} = { }\frac{{E_{h}^{r} }}{{E_{h}^{i} }} = { }\frac{{n_{1} \cos \vartheta_{i} - n_{2} \cos \vartheta_{t} }}{{n_{1} \cos \vartheta_{i} + n_{2} \cos \vartheta_{t} }}$$

33b $${\text{T}} = {\text{T}}_{h} = { }\frac{{E_{h}^{t} }}{{E_{h}^{i} }} = { }\frac{{2n_{1} \cos \vartheta_{i} }}{{n_{1} \cos \vartheta_{i} + n_{2} \cos \vartheta_{t} }},$$

The unit vectors corresponding to the local vertical and horizontal polarizations for both the reflected and transmitted rays can be determined using these formulas.

34a $${\hat{\mathbf{a}}}_{{hr_{p,q} }} = {\hat{\mathbf{a}}}_{{hi_{p,q} }} ,\user2{ } {\hat{\mathbf{a}}}_{{vr_{p,q} }} = {\hat{\mathbf{a}}}_{{kr_{p,q} }} \times {\hat{\mathbf{a}}}_{{hr_{p,q} }}$$

34b $${\hat{\mathbf{a}}}_{{ht_{p,q} }} = {\hat{\mathbf{a}}}_{{hi_{p,q} }} ,\user2{ } {\hat{\mathbf{a}}}_{{vt_{p,q} }} = {\hat{\mathbf{a}}}_{{kt_{p,q} }} \times {\hat{\mathbf{a}}}_{{ht_{p,q} }}$$

The electric field vectors for both the reflected and transmitted rays at the vertex ${\mathbf{V}}_{p,q}$ can be written as follows:

35a $${\mathbf{E}}^{r} = E_{h}^{r} \user2{ }{\hat{\mathbf{a}}}_{{hr_{p,q} }} + E_{v}^{r} \user2{ }{\hat{\mathbf{a}}}_{{vr_{p,q} }}$$

35b $${\mathbf{E}}^{t} = E_{h}^{t} \user2{ }{\hat{\mathbf{a}}}_{{ht_{p,q} }} + E_{v}^{t} \user2{ }{\hat{\mathbf{a}}}_{{vt_{p,q} }}$$

The incident electric field typically includes both vertical and horizontal polarization components. By applying Eqs. (32) and (33) to Eq. (38), one gets:

36a $${\mathbf{E}}^{r} = {\Gamma }_{h} E_{h}^{i} \user2{ }{\hat{\mathbf{a}}}_{{hr_{p,q} }} + {\Gamma }_{v} E_{v}^{i} \user2{ }{\hat{\mathbf{a}}}_{{vr_{p,q} }}$$

36b $${\mathbf{E}}^{t} = {\text{T}}_{h} E_{h}^{i} \user2{ }{\hat{\mathbf{a}}}_{{ht_{p,q} }} + {\text{T}}_{v} E_{v}^{i} \user2{ }{\hat{\mathbf{a}}}_{{vt_{p,q} }}$$

where ${\mathbf{E}}^{r}$ is the reflected field vector and ${\mathbf{E}}^{t}$ is the transmitted field vector. In this scenario, the reflective property at the specific vertex ${\mathbf{V}}_{p,q}$ is determined using the equation provided below

37 $$E^{r} = E_{h}^{r} \cos \psi^{r} + E_{v}^{r} \sin \psi^{r} = {\Gamma }_{h} E_{h}^{i} \cos \psi^{r} + {\Gamma }_{v} E_{v}^{i} \sin \psi^{r}$$

here ${\psi }^{r}$ is the measured separation angle between the reflected electromagnetic field vector and the unit vector aligned with the horizontal direction, and it is given by:

38a $$\psi_{p,q}^{r} = \tan^{ - 1} \left( {\frac{{\left| {E_{v}^{r} } \right|}}{{\left| {E_{h}^{r} } \right|}}} \right) = {\text{tan}}^{ - 1} \left( {\frac{{\left| {{\Gamma }_{v} E_{v}^{i} } \right|}}{{\left| {{\Gamma }_{h} E_{h}^{i} } \right|}}} \right)$$

The angle ${\psi }_{p,q}^{i}$ represents the angular measurement between the incoming electric field vector and the unit vector ${\mathbf{\hat{a}}}_{{h_{{i_{{p,q}} }} }} ,$​

38b $$\psi_{p,q}^{i} = \tan^{ - 1} \left( {\frac{{\left| {E_{v}^{i} } \right|}}{{\left| {E_{h}^{i} } \right|}}} \right)$$

Therefore,

39 $${\Gamma } = \frac{{E^{r} }}{{E^{i} }} = \frac{{{\Gamma }_{h} E_{h}^{i} \cos \psi_{p,q}^{r} + {\Gamma }_{v} E_{v}^{i} \sin \psi_{p,q}^{r} }}{{ E_{h}^{i} \cos \psi_{p,q}^{i} + E_{v}^{i} \sin \psi_{p,q}^{i} }}$$

#### Multiple reflections and higher order scattering

**Fig. 4:**
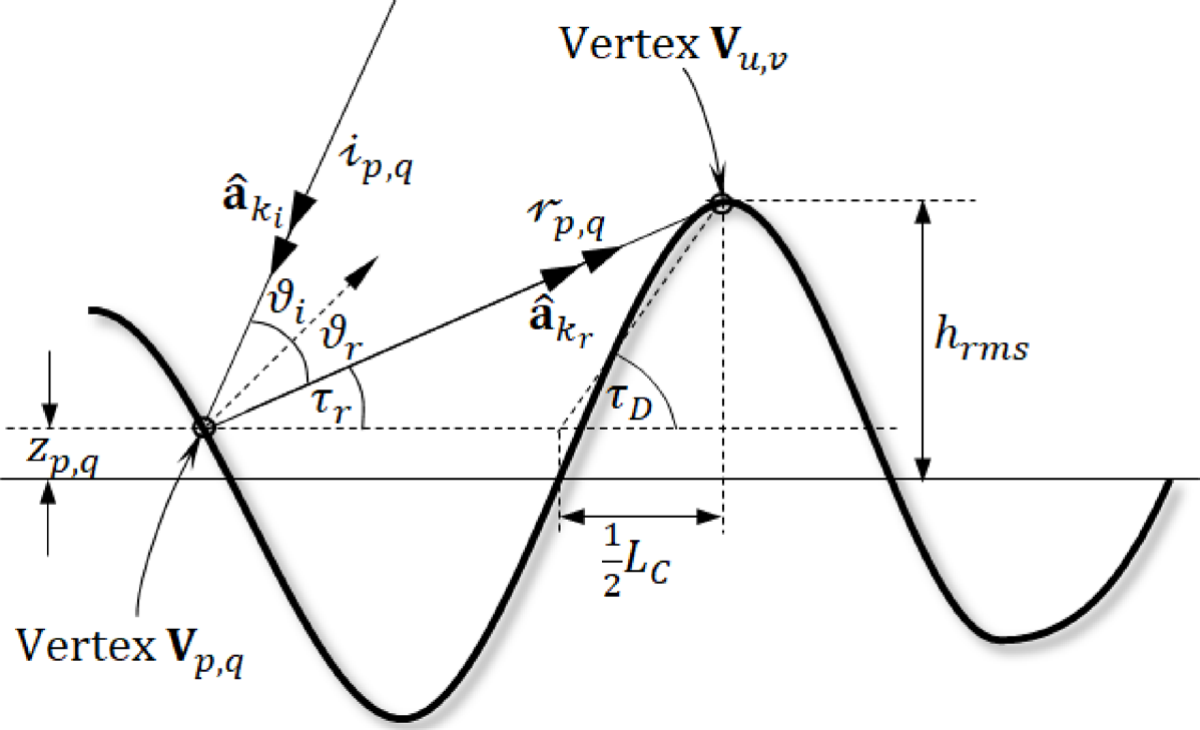
A higher-order reflection of an incident ray is considered only if the reflected ray satisfies the condition ${\tau }_{r}<{\tau }_{D}$.

Initially any ray strikes a point on a rough surface may undergo multiple reflections at other vertices. If a ray hits vertex ${\mathbf{V}}_{p,q}$, it can reflect and collide with the surface again at a different vertex. Figure 4 illustrates the conditions necessary for this reflected ray to intersect the surface. The reflected ray makes an angle ${\tau }_{r}$ with the horizontal direction at the striking point ${\mathbf{V}}_{p,q}.$

40 $$\tau_{r} = \frac{\pi }{2} - \cos^{ - 1} k_{{r_{z} }}$$

In Fig. 4, the reflected ray will avoid further hits with the surface if ${\tau }_{r}>{\tau }_{D}$, ${\tau }_{D}$ is given by

41 $$\tau_{D} = \tan^{ - 1} \left( {R_{D} - \frac{{2z_{p,q} }}{{L_{C} }}} \right)$$

Therefore, only the reflected ray meeting the following requirement is likely to intersect another location on the uneven surface before moving to the distant region, leading to second-order scattering.

42 $$\tau_{r} < \tau_{D}$$

To reduce computational cost, reflected rays that do not meet condition (42) will be excluded from further checks for additional reflections on the uneven surface.

Equation (42) indicates that increased surface roughness leads to more rays undergoing higher-order reflections. The following steps are applied only to the reflected rays that meet this condition.

A ray experiencing its ${t}^{\text{th}}$ bounce at vertex ${\mathbf{V}}_{p,q}$ will intersect the surface at point ${\mathbf{V}}_{u,v}$ if,

43 $${\hat{\mathbf{a}}}_{{k_{r} }}^{\left( t \right)} \cdot {\hat{\mathbf{a}}}_{p,q}^{u,v} = 1 ,$$

Here, ${\widehat{\mathbf{a}}}_{{k}_{r}}^{(t)}$​ represents a normalized directional vector corresponding to the path of the ray after the ${t}^{\text{th}}$ reflection, and ${\widehat{{\varvec{a}}}}_{p,q}^{u,v}$ ​ denotes the unit vector pointing from vertex ${\widehat{{\varvec{a}}}}_{p,q}^{u,v}$ to vertex ${\mathbf{V}}_{u,v}$.

44 $${\hat{\mathbf{a}}}_{p,q}^{u,v} = \frac{{{\mathbf{V}}_{u,v} - {\mathbf{V}}_{p,q} }}{{\left| {{\mathbf{V}}_{u,v} - {\mathbf{V}}_{p,q} } \right|}}$$

**Fig. 5:**
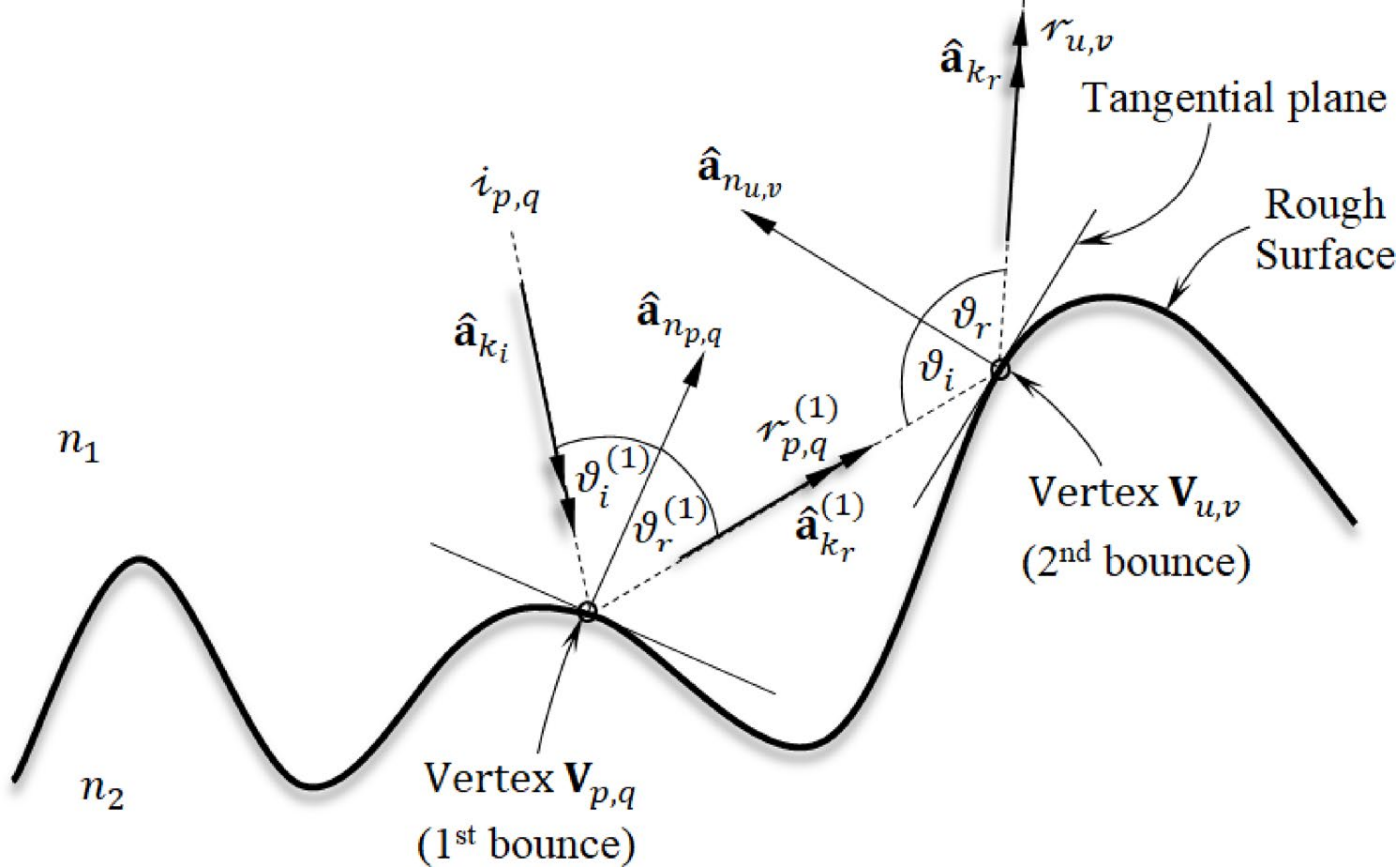
A ray undergoes two bounces at vertices ${\mathbf{V}}_{p,q}$ and ${\mathbf{V}}_{u,v}$ on the rough surface.

If condition (42) is met, the ray will undergo its ${(t+1)}^{\text{th}}$ bounce at vertex ${\mathbf{V}}_{u,v}$ ​, as illustrated in Fig. 5. For every bounce, the trajectory along which the light or electromagnetic wave propagates after bouncing off a surface is determined and explained in "Directions and phases of reflected rays" section. The Fresnel reflection parameters corresponding to the specific locations where the wave encounters the surface are computed according to "Fresnel law at the interface" section. The phase of a wave propagating into the far-field region can subsequently be determined using the following calculation:

45 $$\Delta \Phi _{{p,q}} = \frac{{2\pi }}{\lambda }\left( {~\Delta _{{i_{{p,q}} }} - ~\Delta _{{r_{{u,v}} }} } \right) + \mathop \sum \limits_{{\nu = 2}}^{{N_{{O_{{p,q}} }} }} \frac{{2\pi }}{\lambda }D^{{\left( {\nu - 1} \right)}}$$

In this case, ${D}^{(\nu -1)}$ refers to the distance the reflected ray covers between the ${\left(\nu -1\right)}^{\text{th}}$ and ${\nu }^{\text{th}}$ impact locations on the surface, starting at ${\left(\nu -1\right)}^{\text{th}}$ point, with $\nu \ge 2$. ${{N}_{O}}_{p,q}$ represents the maximum number of bounces taken into account, which matches the order of the GTD-RT method.

### Distribution of deflected optical beams on irregular surfaces

**Fig. 6:**
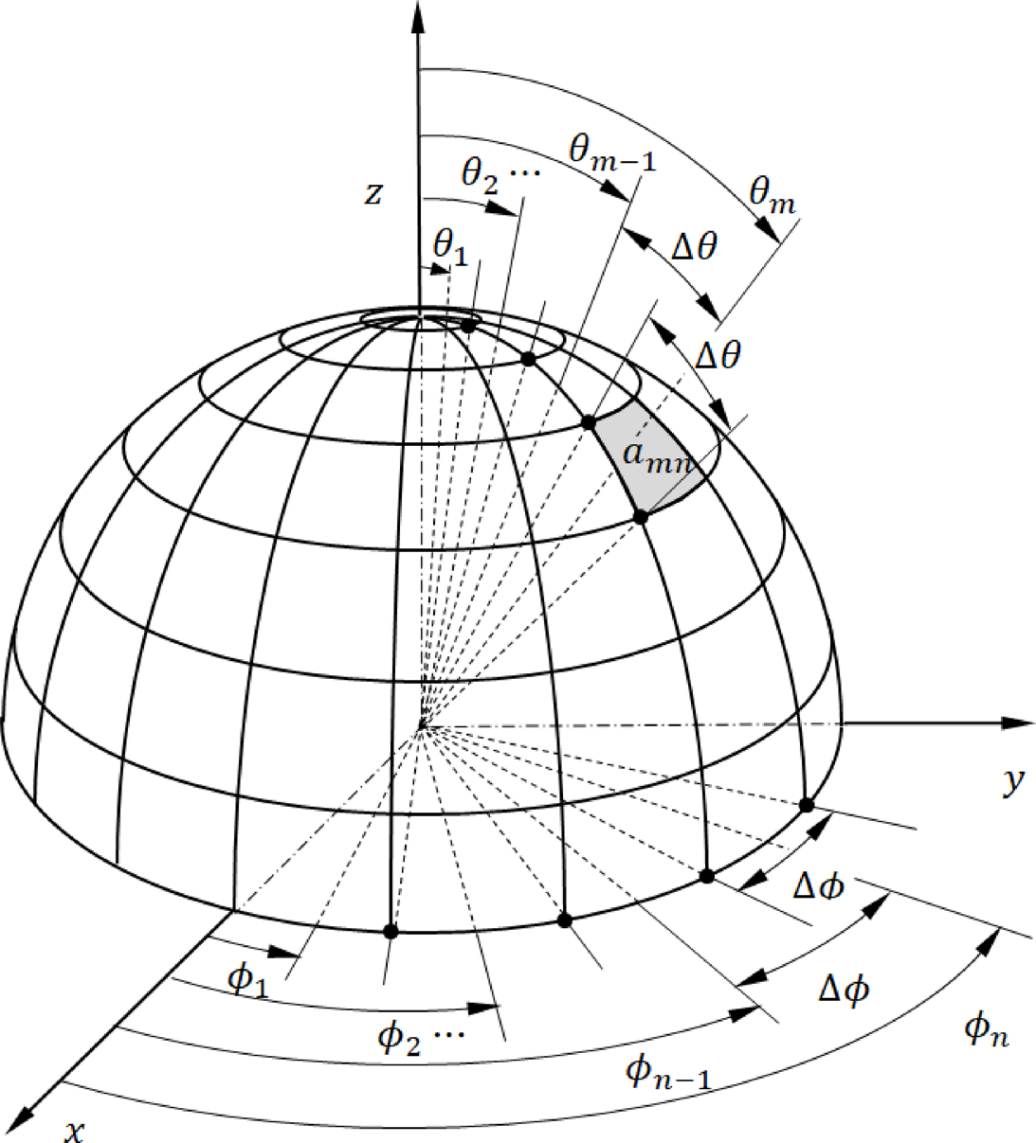
A divided curved boundary of a half-sphere placed over the irregular surface, where its mean latitude (zero) lies within in $xy-$ plane.

Imagine a vast semi-spherical region with an infinite radius $R\to \infty$, extending across the entire upper hemispherical region (where $z>0$), as depicted in Fig. 6. The boundary of this semi-spherical region is divided evenly in $\theta$- and $\phi$, creating $M\times N$ spherical surface segments, with their areas calculated as follows.

46 $$a_{mn} \approx R^{2} {\text{ sin}}\theta_{m} { }\Delta \theta { }\Delta \phi ,{ }m = 1,2, \ldots ,M,{ }n = 1,2, \ldots ,{ }N$$

47 $$\Delta \theta = \frac{\pi }{2M},{ }\Delta \phi = \frac{2\pi }{N}$$

48 $$\theta_{m} = \frac{2m - 1}{2}\Delta \theta ,{ }m = 1,2, \ldots ,M,{ }\phi_{n} = \frac{2n - 1}{2}\Delta \phi ,{ }n = 1,2, \ldots ,{ }N$$

By substituting Eq. (47) into Eq. (46), the result is:

49 $$a_{mn} \approx { }\frac{{\pi^{2} }}{MN}{ }R^{2} {\text{ sin}}\theta_{m} ,{ }m = 1,2, \ldots ,M,{ }n = 1,2, \ldots ,{ }N$$

Take ${Q}_{i}$ parallel rays incident at $\left({\theta }_{i},{\phi }_{i}=0\right)$, evenly spread across the surface as outlined in Section number 3. To properly evaluate the distribution of scattered waves throughout the upper hemispherical region, the condition below shall be satisfied:

50 $$Q_{i} > > MN$$

The points, generated according to the methodology outlined in “Rough surface modeling” section for the irregular surface representation, are joined by three-sided segments (triangles). Every individual beam ${\mathcalligra{i}}_{p,q}$ hitting vertex ${\mathbf{V}}_{p,q}$ will reflect, unless obstructed by another portion of the rough surface. Consequently, the total number of reflected rays ${Q}_{r}$, is less than or equal to the number of incident rays ${Q}_{i}$​.

51 $$Q_{r} \le Q_{i}$$

The direction of the reflected ray is governed by snell’s law of reflection and is determined by the position-specific directional vector indicating the perpendicular vector to the surface at the specified vertex, which corresponds to the perpendicular vector at the specified location of impact on the rough surface.

Let’s specify ${\psi }_{p,q}$ of the returning ray ${\mathcalligra{r}}_{p,q}$ as the combination of two angles that describe the ray’s trajectory after it reflects, typically involving the angle ${\theta }_{p,q}$ and the angle ${\phi }_{p,q}$, which indicate the ray’s position in relation to a reference axis.

52 $${ }\psi_{p,q} { } \equiv \left( {\theta_{p,q} ,\phi_{p,q} } \right)$$

Here, ${\theta }_{p,q}$ and ${\phi }_{p,q}$​ denote the angles between the reflected ray ${\mathcalligra{r}}_{p,q}$ and the positive directions of the global $z$ and $x$ axes, correspondingly.

Let, ${\Psi }_{m,n}$ be defined as,

53 $${{ \Psi }}_{m,n} \equiv \left\{ {\left( {\theta ,\phi } \right): \theta_{n} - \frac{1}{2}\Delta \theta \le \theta < \theta_{n} + \frac{1}{2}\Delta \phi , \phi_{n} - \frac{1}{2}\Delta \phi \le \phi < \phi_{n} + \frac{1}{2}\Delta \phi } \right\}$$

Let the returning ray ${r}_{p,q}$ has a power density which could represented by.

54 $$\frac{{\mathcalligra{p}_{{r_{{p,q}} }} }}{{a_{{m,n}} }} = \frac{1}{{2\eta _{o} }}\left| {E_{{r_{{p,q}} }} } \right|^{2}$$

here, ${\eta }_{o}$ is the characteristic electromagnetic resistance of a vacuum 377 Ω, and $\left|{{E}_{r}}_{p,q}\right|$ denotes the absolute value of electromagnetic force field related to ${r}_{p,q}$​. By applying Eq. (23), we obtain:

55 $$\frac{{\left| {\Gamma _{{p,q}} } \right|^{2} ~\mathcalligra{p}_{{i_{{p,q}} }} }}{{a_{{m,n}} }} = \frac{1}{{2\eta _{o} }}\left| {E_{{r_{{p,q}} }} } \right|^{2}$$

where,

56 $$\frac{{\left| {{{\Gamma }}_{{p,q}} } \right|^{2} \mathcalligra{p}_{{i_{{p,q}} }} }}{{a_{{m,n}} }} = \frac{1}{{2\eta _{o} }}\left| {E_{{r_{{p,q}} }} } \right|^{2}$$

Here, ${\Gamma }^{\left(\nu \right)}$ represents the Fresnel reflection coefficient of ray ${\mathcalligra{i}}_{p,q}$, which first strikes vertex ${\mathbf{V}}_{p,q}$​, at the ${\nu }^{th}$ bounce. ${{N}_{O}}_{p,q}$ indicates the total number of bounces that ray ${\mathcalligra{i}}_{p,q}$ undergoes. Note that ${\Gamma }^{\left(\nu \right)}$ is determined using Eqs. (32), (33), or generally (39), by assigning $u$ and $v$ to the coordinate identifiers of the point where ${\nu }^{\text{th}}$ reflection happens. Therefore, the dispersed electromagnetic field in the far-field region shall be computed by:

57 $$E_{{mn}} = E\left( {\theta _{m} ,\phi _{n} } \right) = e^{{ - jk_{o} R}} \mathop \sum \limits_{{\psi _{{p,q}} ~ \in ~\Psi _{{m,n}} }} \Upsilon _{{p,q}} ~\Gamma _{{p,q}} ~~\sqrt {\frac{{2\eta _{o} ~\mathcalligra{p}_{{i_{{p,q}} }} }}{{a_{{m,n}} }}} ~e^{{ - j~\frac{{2\pi }}{\lambda }~\Delta \Phi _{{p,q}} }}$$

Using Eqs. (48) and (55), the final expression can be written as:

58 $$E_{{mn}} = ~~\frac{{e^{{ - jk_{o} R~}} }}{R}~\frac{{\sqrt {MN} }}{\pi }~\sqrt {\frac{{\eta _{o} }}{{\sin \theta _{m} }}} ~~\mathop \sum \limits_{{\psi _{{p,q}} ~ \in ~\Psi _{{m,n}} }} \Upsilon _{{p,q}} ~\mathop \prod \limits_{{\nu = 1}}^{{N_{{O_{{p,q}} }} }} \Gamma ^{{\left( \nu \right)}} ~\sqrt {\mathcalligra{p}_{{i_{{p,q}} }} } ~e^{{ - j~\frac{{2\pi }}{\lambda }~\Delta \Phi _{{p,q}} }}$$

## Results and discussions

This section investigates the reliability GTD-RT method introduces with higher-order considerations for evaluating scattering when an optical signal hits a rough surface. The reflection and transmission scattering patterns are computed. It looks into how using higher-order GTD-RT improves result precision, comparing patterns derived from second-order and first-order approaches. The study also explores how the surface roughness affects the scattering patterns. Furthermore, the role of the incidence wave polarization is investigated. Finally, the obtained scattering pattern numerically using the proposed technique is compared experimentally with a measured data of a rough surface with known properties.

### Interaction of light with rough surfaces: reflection and transmission

**Fig. 7:**
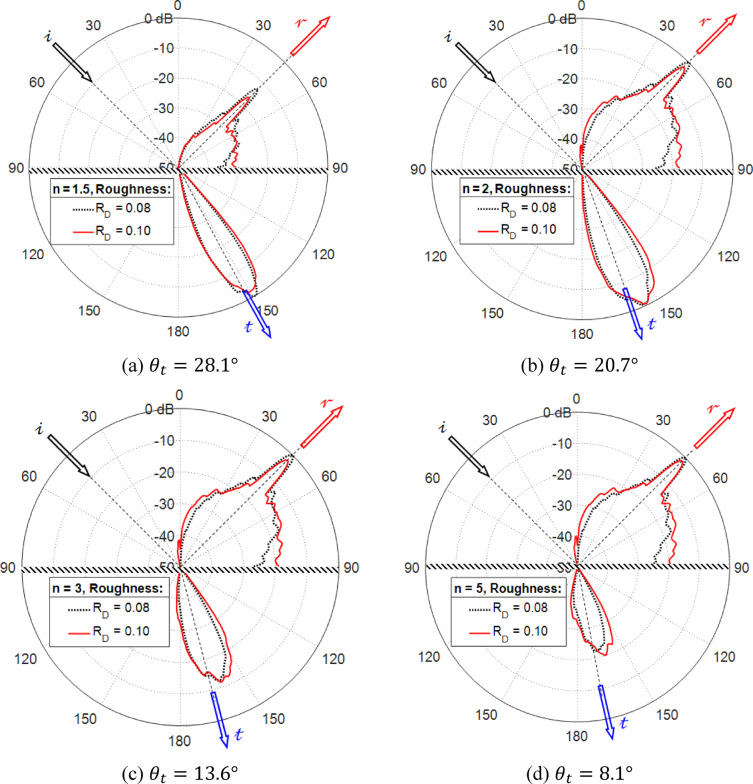
The distribution patterns of a plane wave as it undergoes both reflection and transmission with a wavelength of $\lambda =1000 \text{nm}$ are examined on rough surfaces of size (20 μm × 20 μm), with a correlation length of ${L}_{C}=2\,{ \upmu m}$ and a resolution of 1000 × 1000 points, for varying refractive indices. The incident angle is ${\theta }_{i}^{(n)}=45^\circ$. The transmission direction, based on Snell’s law, is indicated by the angle ${\theta }_{t}$ for each scenario.

The approach used in this study calculates both the portion of electromagnetic energy redirected into the upper hemispherical region and the fraction that propagates into the lower hemispherical region. Figure 7 illustrates the distribution of scattered waves, encompassing both reflected and transmitted components, resulting from a plane wave striking flat horizontal surfaces with relatively low roughness (${R}_{D}=0.08, 0.10$). The plane wave strikes at a 45° angle relative to the upright direction, as shown by the arrow marked $\mathcalligra{i}$. According to Snell’s law, the path of the specularly reflected wave is indicated by the arrow $\mathcalligra{r}$, while the path of specular transmitted (refracted) wave is marked by the direction $\mathcalligra{t}$. The results indicate: as a result of the surface’s slight roughness, both the reflected and transmitted waves exhibit mostly specular behavior, with very little diffusion of scattered waves. Additionally, it is observed that a higher refractive index leads to an increase in reflected power while reducing the transmitted power. At lower refractive indices ($n=1.5$), the power of the transmitted ray is significantly greater than the power in the reflected ray as depicted in Fig. 7a. In contrast, Fig. 7d demonstrates that at higher refractive indices ($n=5$), the power of reflection is substantially greater than the power of the transmission.

### Evaluation of the precision of GTD-RT technique with first and second-order

**Fig. 8:**
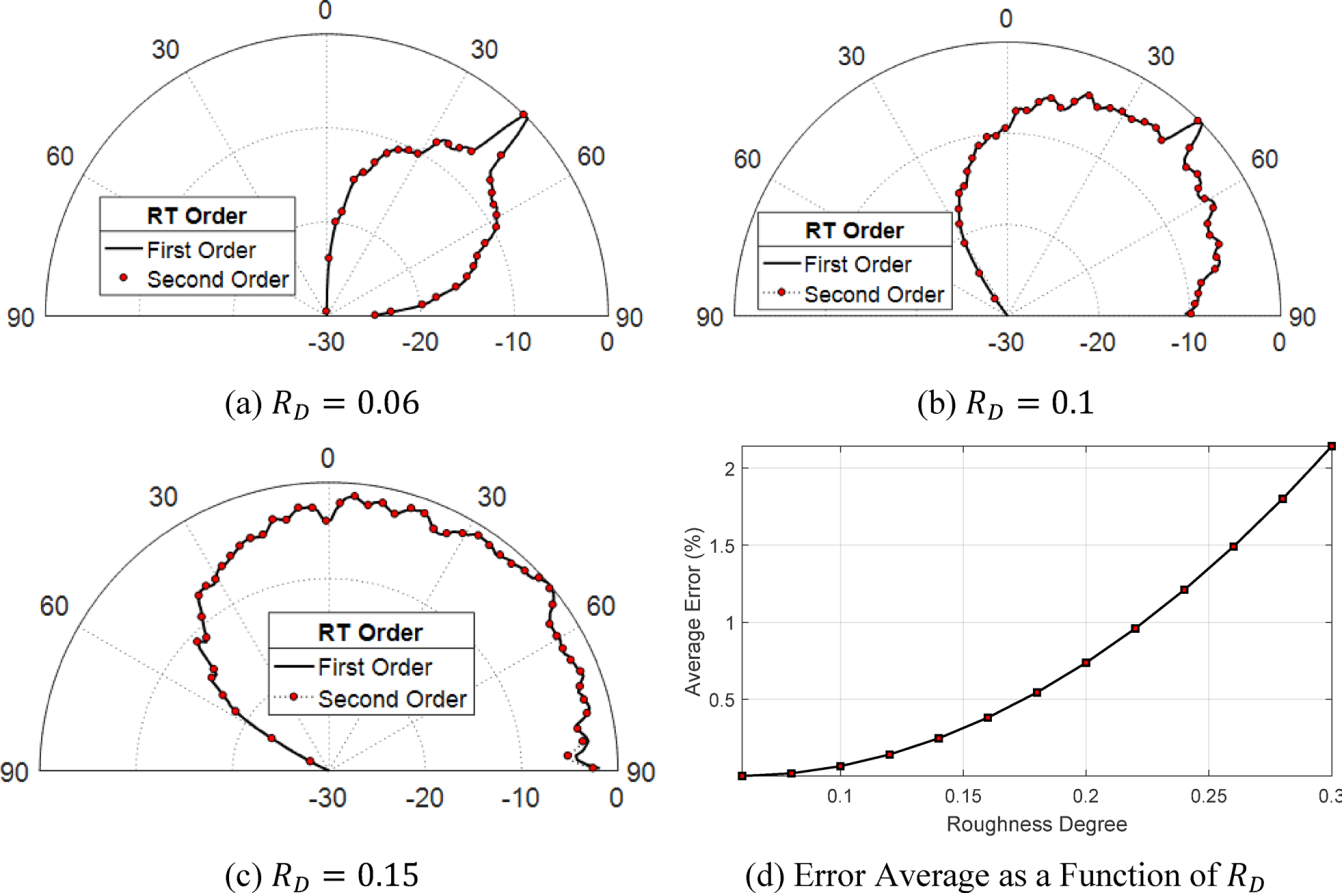
The scattering of a plane wave with vertical polarization and a wavelength of λ = 635 nm, striking a rough surfaces with a correlation length of ${L}_{C}=7.16$ μm and an incident angle of ${\theta }_{i}^{(n)}=45^\circ$, is evaluated using both first-order and second-order GTD-RT. The surface models have a resolution of 2000 × 2000 vertices.

The second-order GTD-RT method demands significantly more numerical resources than the first-order GTD-RT method. This section aims to assess the enhancement in the results achieved by employing second-order GTD-RT compared to the first-order approach. To achieve this, three sets of rough surface representations are created with diverse statistical properties. Each set consists of 20 different rough surface samples generated with identical characteristics. The surface models categorized into the first, second, and third sets are characterized by roughness of $RD = 0.06$, 0.10, and 0.15, respectively. Each of these models has been designed with a fixed correlation length of ${L}_{C}=7.16$ µm, ensuring consistency in surface feature distribution while varying the degree of roughness across the different sets. Figure 8a,b,c illustrate the scattering distributions that result from a plane wave striking the surface at a 45° angle to the surface normal for each set of the three sets. These patterns provide insights into how the wave interacts with different roughness levels, influencing both reflection and transmission behaviors. It is evident that, in all cases, the improvement from applying the second-order GTD-RT method is minimal, even for surfaces with higher roughness. Although the second-order method yields more precise results and offers a higher level of accuracy compared to the first-order approach, there remains a measurable discrepancy between the results produced by the two techniques. The average error in the scattering coefficients obtained through first-order ray tracing can be quantitatively defined as the difference between the mean scattering coefficients calculated using the first-order method and the corresponding values derived from the more precise second-order method. This error is evaluated by considering all possible directional components across the entire half-space, providing a comprehensive measure of the deviation introduced by the lower-order approximation. The average percentage error is determined by evaluating the proportion of the computed difference between the first-order and second-order scattering coefficients relative to the mean scattering coefficients derived from the second-order ray tracing method. This ratio provides a quantitative measure of the deviation introduced by the first-order approximation. The calculation of this error follows a specific mathematical formulation, which is expressed as

59 $${\text{Average error }}\left( {\text{\% }} \right) = \mathop \sum \limits_{\theta ,\phi }^{{}} \frac{{\left| {E_{{1^{{{\text{st}}}} }}^{r} \left( {\theta ,\phi } \right) - E_{{2^{{{\text{nd}}}} }}^{r} \left( {\theta ,\phi } \right)} \right|}}{{\left| {E_{{2^{{{\text{nd}}}} }}^{r} \left( {\theta ,\phi } \right)} \right|}} \times 100\%$$

here, ${E}_{{1}^{\text{st}}}^{r}(\theta ,\phi )$ and ${E}_{{2}^{\text{nd}}}^{r}(\theta ,\phi )$ represent the scattered electromagnetic fields propagating along the specified direction $(\theta ,\phi )$ computed using first and second-order ray tracing methods, respectively.

Figure 8d presents a visualization of the relationship between average percent error and surface roughness. The data clearly demonstrate that as the roughness of the surface increases, the discrepancy introduced by employing the first-order method instead of the more precise second-order method becomes more pronounced. This trend highlights the growing limitations of the first-order approach in accurately capturing scattering behavior on increasingly rough surfaces. Nonetheless, even when dealing with surfaces exhibiting substantial roughness, such as those with an RD value of 0.3, the average percentage error remains relatively low, staying under 2.5%. This indicates that despite the increased complexity introduced by rougher surfaces, the first-order method maintains a reasonable level of accuracy. Given that second-order and higher-order GTD-RT techniques require significantly greater computational resources, the marginal enhancement in precision they offer does not always justify the additional computational cost. As a result, the first-order approach emerges as a more practical and efficient option for analyzing optical scattering from irregular surfaces, particularly in scenarios where computational efficiency is a priority.

### Effect of surface roughness on the scattering pattern

**Fig. 9:**
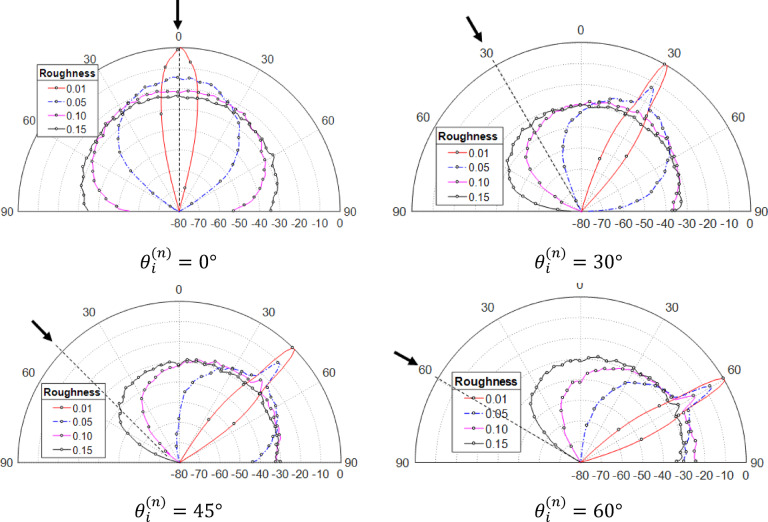
Patterns of a vertical polarized wave striking surface with a 1000 × 1000 vertex resolution and a correlation length ${L}_{C}=7.16\,{ \upmu m}$, for different roughness levels and incident angles.

The distribution of scattered waves from rough surfaces changes depending on the level of surface roughness. This variation is depicted in Fig. 9, which illustrates how the scattering pattern is influenced by different angles of incidence. As roughness increases, the scattered beam widens. For low roughness (RD = 0.01), scattering is mostly specular with little diffuse scattering. As the surface roughness increases, with RD values of 0.05 and 0.1, the proportion of diffuse scattering becomes more prominent, while the intensity of specular scattering diminishes. This shift indicates that as the surface becomes more irregular, the scattered waves are dispersed in a wider range of directions rather than being concentrated in a single reflected direction, leading to a broader beam and weaker specular peak. At high roughness (RD = 0.15), scattering becomes almost entirely diffuse. The angle of incidence also affects the scattering pattern, with a stronger specular component and reduced backscatter at higher incidence angles. Overall, higher roughness leads to more backscattering, regardless of the incidence angle ${\theta }_{i}^{(n)}.$

### Effect of incident field polarization on scattering

**Fig. 10:**
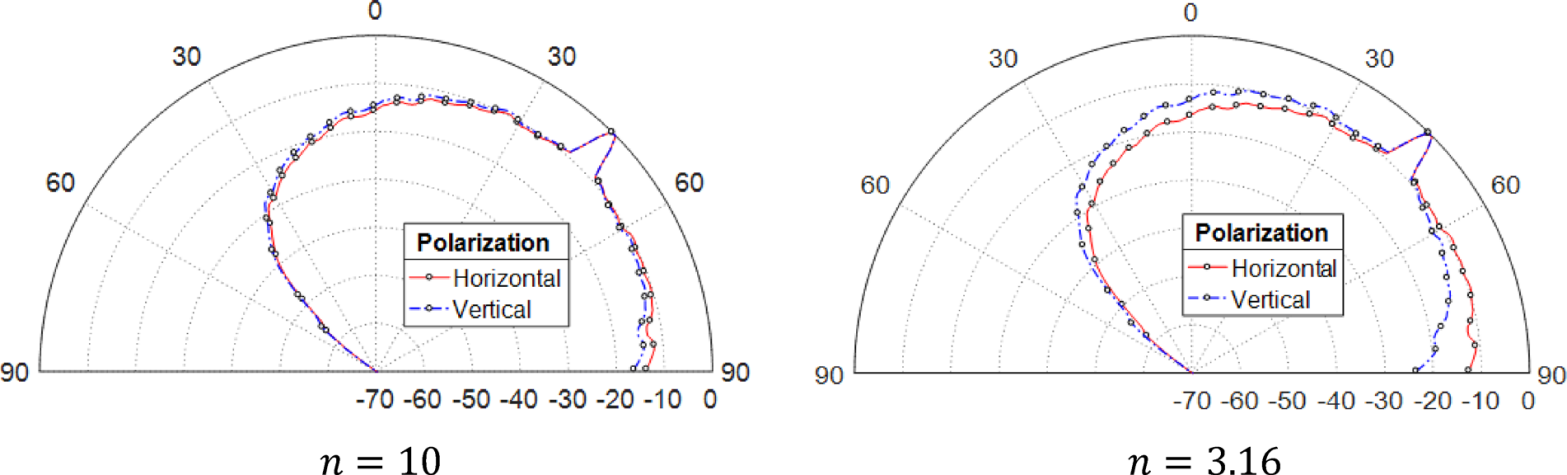
Patterns of a vertical and horizontal polarized wave striking surface 63.5 × 63.5 μm at an incident angle of ${\theta }_{i}^{(n)}=45^\circ$, with a resolution of 1500 × 1500 points, roughness factor ${R}_{D}=0.10$, wavelength $\lambda =635$ nm, and correlation length ${L}_{C}=7.16$ μm.

**Fig. 11:**
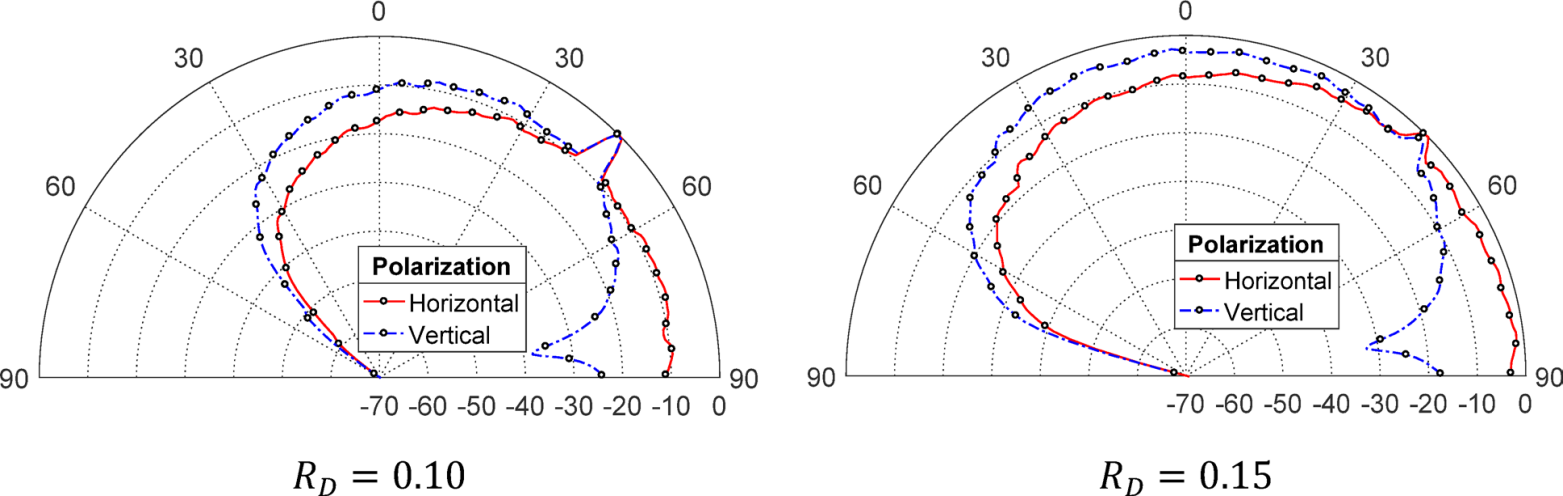
Patterns of a vertical and horizontal polarized wave striking surface 63.5 × 63.5 μm rough surface at an incident angle of ${\theta }_{i}^{(n)}=45^\circ$, with a resolution of 1500 × 1500 points, wavelength *λ* = 635 nm, correlation length ${L}_{C}=7.16$ μm, and refractive index *n* = 1.5.

The scattering characteristics of both horizontal and vertical polarized waves, which strike a rough surface functioning as the boundary between air and a dielectric material, are investigated under varying levels of surface roughness and different refractive indices. This analysis provides insights into how polarization influences wave behavior when interacting with an irregular interface. As demonstrated in Fig. 10a, when the refractive index is considerably high ($n = 10$), the scattering patterns corresponding to both H-polarized and V-polarized waves exhibit a strong resemblance. This observation indicates that as the refractive index increases, the impact of polarization on the scattering process diminishes, resulting in nearly identical scattering distributions for both types of waves. Such behavior suggests that at higher refractive indices, the rough surface scatters incoming waves in a manner that is largely independent of their polarization state, simplifying the overall scattering analysis. However, at lower refractive indices ($n = 3.16$, $n = 1.5$), illustrated in Figs. 10b, 11a and b, the scattering patterns for the two polarizations become distinct. A decrease in the refractive index leads to a rise in backscattering for the V-polarized wave, while simultaneously amplifying forward scattering for the H-polarized wave along the direction parallel to the surface. This shift in scattering behavior highlights the influence of refractive index variations on wave propagation and directional energy distribution. Furthermore, as the surface roughness increases, the specular scattering decreases, with more diffuse scattering occurring for both polarization types, as illustrated in the comparison of the patterns in Fig. 11a and b.

### Experimental evaluation

**Fig. 12:**
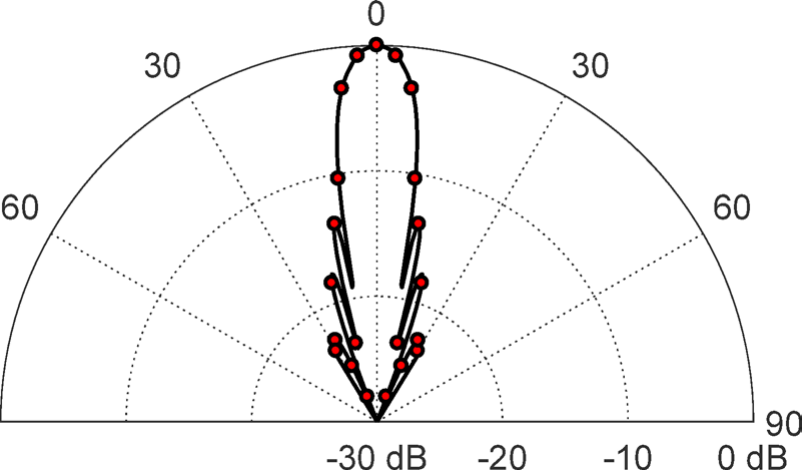
The distribution of radiation pattern of the light beam utilized for experimental observations, $\lambda =635$ nm.

**Fig. 13:**
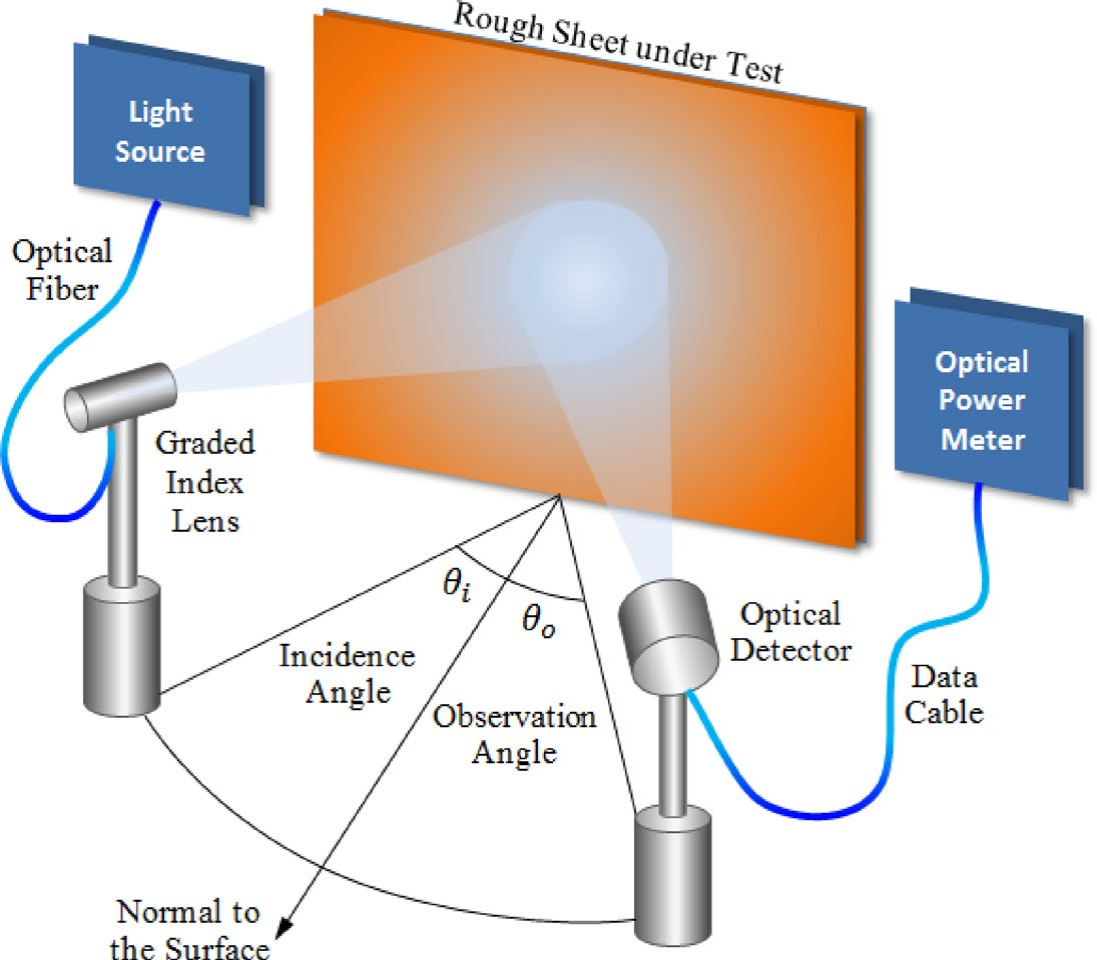
The experimental setup for observing the scattering pattern produced by a light beam striking a rough surface utilizes a 635 nm laser source and a Thorlabs® optical power meter (model PM100) paired with an optical detector (model S120B).

The reliability of the GTD-RT approach formulated in this study for determining the far-field scattering pattern from rough surfaces with arbitrary statistical characteristics is assessed by comparing its predictions with empirical observations. To validate the method, an incident plane wave is produced using a light source operating at a wavelength of λ = 635 nm. The radiation pattern emitted by this source is captured and analyzed, as illustrated in Fig. 12, utilizing a Thorlabs® PM100 optical power meter in conjunction with an S120B optical sensor. The experimental setup designed to measure the scattering pattern is outlined in Fig. 13. In this configuration, the rough surface is securely fixed onto a vertical wall, while the light source is carefully aligned so that the incoming beam strikes the surface at a 45° angle relative to the normal. To systematically record the intensity of the scattered light, an optical sensor, connected to the power meter, is rotated across a wide angular range from − 90° to 90° with respect to the surface normal. To thoroughly assess the accuracy of the proposed method under different surface conditions, two distinct types of white sheets with varying degrees of roughness are employed in the experiment. The first is a glossy white sheet characterized by a roughness parameter of ${R}_{D}=0.09,$ while the second is a matte white sheet with a significantly higher roughness level of ${R}_{D}=0.225.$ These variations in surface texture enable a comprehensive evaluation of the GTD-RT model’s effectiveness in predicting optical scattering behavior across different roughness conditions.

**Fig. 14:**
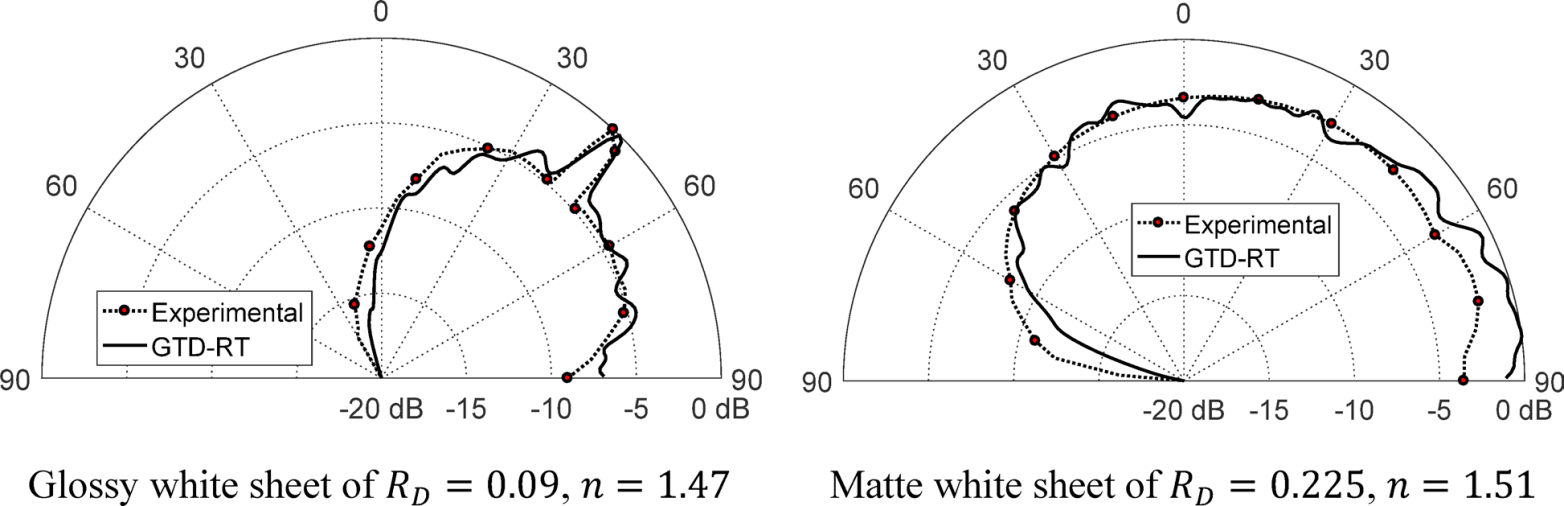
A comparison is made between the scattering patterns acquired from experimental measurements and those calculated using the GTD-RT method for an optical wave with vertical polarization and wavelength of $\lambda =635$ nm striking flat white sheets with varying roughness levels. The correlation length is set to ${L}_{C}=7.16$ μm, and the incident angle is ${\theta }_{i}^{(n)}=45^\circ$.

A smooth flat sheet, characterized by a surface irregularity of ${R}_{D}=0.09$ and an optical refractive index of $n=1.47$, is illuminated by a light beam, as shown in Fig. 8. The resulting scattering distribution of the reflected light is measured using an optical power meter, following the procedure detailed in the experimental setup. The recorded scattering pattern is then evaluated against theoretical predictions generated using the second-order GTD-RT method developed in this study. The comparison between experimental observations and numerical simulations is depicted in Fig. 14a, providing insight into the model’s accuracy in characterizing optical scattering from surfaces with minor roughness variations. Due to the minor irregularities on the reflective surface, the scattering pattern displays a mix of mirror-like (specular) and diffuse reflection characteristics. The close match between the experimental observations and numerical simulations confirms the reliability of the GTD-RT method in predicting plane wave scattering on textured surfaces. The procedure is repeated for another sheet with a surface roughness of ${R}_{D}=0.225$ and a refractive index of $n=1.51$. The resulting scattering distribution is compared with the one computed using the second-order GTD-RT technique, as shown in Fig. 14b. In this scenario, the correlation distance for the surface model is set to ${L}_{C}=7.16$ µm. Both the recorded and simulated scattering distributions show scattered reflection due to the greater surface irregularities. The strong alignment between the measured data and the numerical analysis further supports the accuracy of the proposed GTD-RT model. It is important to mention that the rough sheets used in this optical scattering experiment have surface heights following a Gaussian distribution and a Gaussian correlation function.

## Comparison with existing numerical methods in literature

**Fig. 15:**
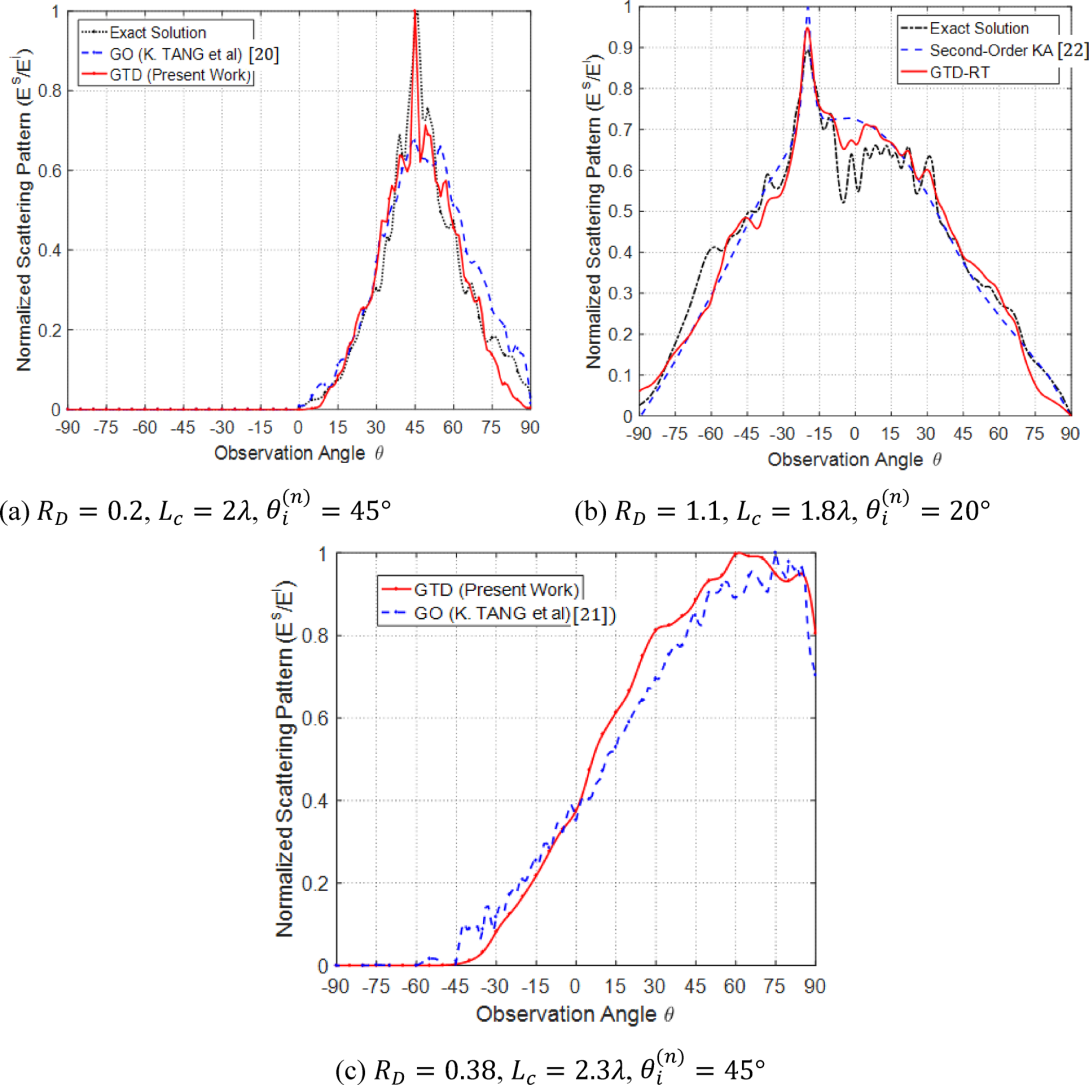
Scattering patterns resulting from a plane wave incident at ${\theta }_{i}^{(n)}=45^\circ$ on a perfectly conducting rough surface of size $100\lambda \times 100\lambda$, discretized into a $1000\times 1000$ mesh, are compared with previously published results.

Scattering coefficients for a rough, perfectly conducting surface (with parameters ${R}_{D}=0.2$ and ${L}_{c}=2\lambda$) are analyzed using the Geometrical Optics (GO) method and benchmarked against an exact electromagnetic solution from^19^. The GO-based results, as detailed in^19^, are shown alongside the exact solution in Fig. 15a. The proposed GTD-RT approach is applied to an ensemble of 10 statistically similar rough surface samples, and the average scattering pattern is evaluated. This new method yields results that align more closely with the exact solution than those obtained via the GO method in^19^.

For a highly rough, perfectly conducting surface characterized by ${R}_{D}=1.1$ and ${L}_{c}=1.8\lambda$, the scattering pattern computed using the proposed GTD-RT method (averaged over 10 surface realizations) is compared with results from^21^ using the modified second-order Kirchhoff approximation, and with the exact solution obtained via Monte Carlo averaging of Method of Moments (MoM) computations. As illustrated in Fig. 15b, the GTD-RT results show better agreement with the exact solution than the Kirchhoff-based approach. For a 2D perfectly conducting rough surface with ${R}_{D}=0.38$ and ${L}_{c}=2.3\lambda$, the scattering pattern generated by the proposed GTD-RT method (averaged over 10 realizations) is compared with the GO-based results from^20^. As illustrated in Fig. 15c, the two approaches exhibit strong agreement.

To conclude the numerical results, it’s helpful to outline the key contributions of this work in comparison to established methods such as Monte Carlo averaging, the Kirchhoff Approximation (KA), and Geometrical Optics (GO). While Monte Carlo techniques provide highly accurate reference solutions by averaging Method of Moments (MoM) results across large ensembles, they are computationally expensive and require hundreds or even thousands of surface realizations to reduce statistical error. In contrast, the proposed GTD-RT method achieves comparable or better accuracy using significantly fewer samples typically between 5 and 10 thanks to a more efficient ray distribution strategy. Despite requiring ensemble averaging like Monte Carlo methods, the GTD-RT approach offers superior computational efficiency and improved accuracy over GO and KA, as demonstrated through several comparative examples in Sect. 4.7.

**Fig. 16:**
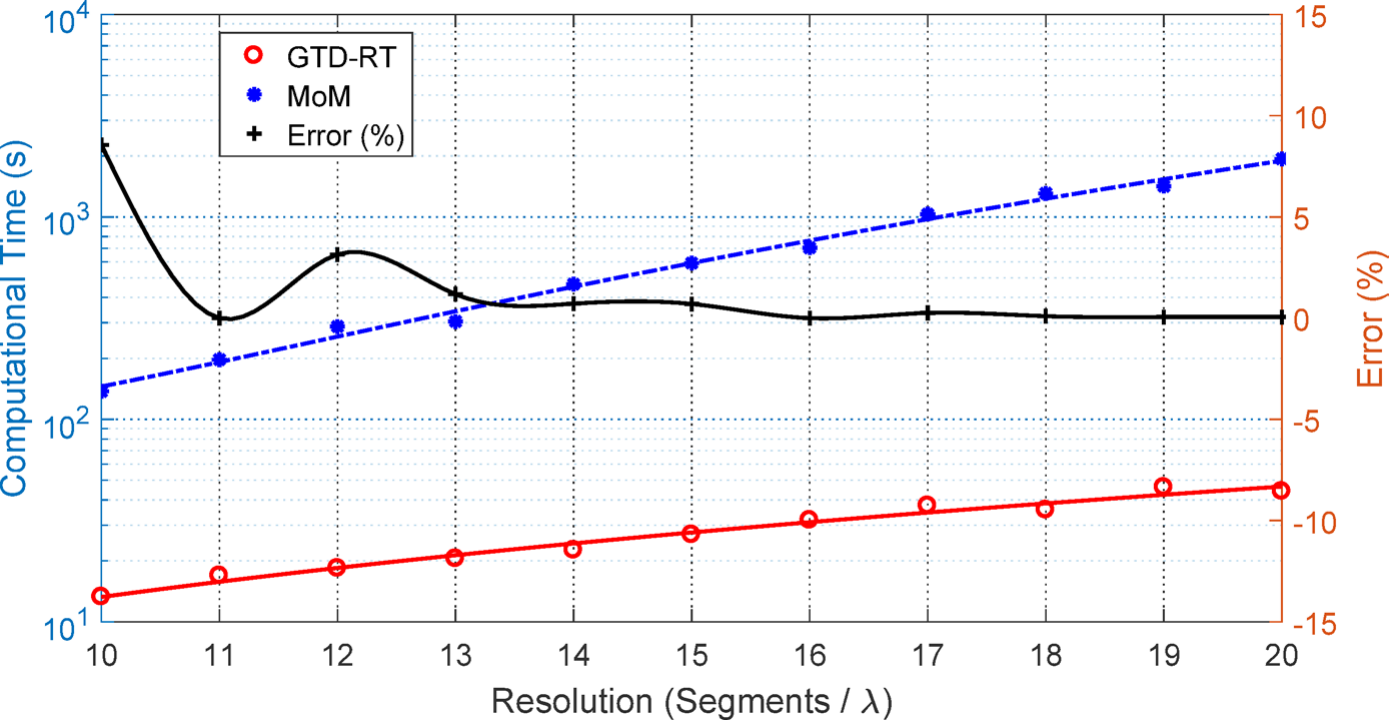
The computational time required by the GTD-RT method is compared to that of the Method of Moments (MoM) for computing scattering patterns and associated errors under plane wave incidence at ${\theta }_{i}^{(n)}=45^\circ$ on a perfectly conducting rough surface of size $10\lambda \times 10\lambda$, with parameters ${R}_{D}=0.38$, ${L}_{c}=2.3\lambda$, and varying surface resolution.

Previous studies using Monte Carlo simulations often rely on highly accurate numerical methods such as the Method of Moments (MoM)^22^, Finite Element (FE), or Finite-Difference Time-Domain (FDTD) to compute scattered fields^23^. The key contribution of the current work lies in achieving both high accuracy and computational efficiency through the proposed GTD-RT algorithm. As demonstrated in Fig. 15, the GTD-RT results closely match the exact solution, surpassing the accuracy of GO and KA methods. Unlike traditional Monte Carlo approaches, which require large ensembles, the GTD-RT method produces reliable results with as few as 5–20 surface realizations. Furthermore, performance comparisons shown in Fig. 16 reveal that the GTD-RT method significantly reduces computation time relative to MoM when applied to the same rough surface scenario.

Given that the Method of Moments (MoM) yields a more accurate scattered field than the GTD-RT approach, the error in the GTD-RT-derived scattering coefficients is defined as the difference between the angular average of the GTD-RT results and that of the MoM. The percentage error is then calculated as the ratio of this difference to the average value from the MoM. This can be expressed mathematically as follows:

61 $${\text{Error }}\left( {\text{\% }} \right) = \mathop \sum \limits_{\theta ,\phi }^{{}} \frac{{\left| {E_{GTD}^{r} \left( {\theta ,\phi } \right) - E_{MoM}^{r} \left( {\theta ,\phi } \right)} \right|}}{{\left| {E_{MoM}^{r} \left( {\theta ,\phi } \right)} \right|}} \times 100\%$$

here, ${E}_{GTD}^{r}\left(\theta ,\phi \right)$ and ${E}_{MoM}^{r}\left(\theta ,\phi \right)$ are the scattered electric fields in the direction $(\theta ,\phi )$ calculated using the GTD-RT and the MoM, respectively. Results indicate that increasing the surface model resolution ($\lambda /\Delta$) leads to a noticeable reduction in the error of the scattered field computed using the GTD-RT method, with the error becoming negligible at sufficiently high resolutions.

## Conclusions

This work presents an enhanced modeling framework that integrates the Geometrical Theory of Diffraction (GTD) with Ray Tracing (RT) techniques to investigate optical scattering from randomly rough surfaces with defined electromagnetic and statistical properties. The approach leverages Fresnel-based formulations to accurately estimate the spatial distribution of scattered power, accounting for both reflection above and transmission below the surface interface. Importantly, the model incorporates the polarization state of the incident wave and supports higher-order interactions, enabling the simulation of multi-bounce scattering phenomena. The performance and validity of the proposed higher-order GTD-RT method were assessed through comparison with experimental scattering data obtained from controlled measurements on paper surfaces exhibiting varying roughness levels and statistical distributions. Numerical simulations further analyzed the effects of surface granularity, angle of incidence, and refractive index on the resulting scattering profiles. By calculating and comparing both first- and second-order scattering contributions, the second-order GTD-RT method provides slightly improved accuracy over the first-order method, especially for highly rough surfaces, but the enhancement remains marginal. Given its low average error (< 2.5%) and significantly lower computational cost, the first-order GTD-RT method offers a more practical and efficient solution for optical scattering analysis in rough surface scenarios. To further verify the credibility of the method, selected outcomes were benchmarked against well-established theoretical models such as Geometrical Optics (GO) and the second-order Kirchhoff approximation. The consistency between the proposed model and these reference approaches reinforces its applicability and robustness in capturing the physical behaviour of light scattering from rough dielectric interfaces. Overall, the presented methodology offers a practical and accurate tool for optical wireless communication system analysis, particularly in indoor environments where surface-induced scattering significantly impacts signal integrity.

## Data Availability

The datasets used and/or analyzed during the current study available from the corresponding author on reasonable request.

## References

[1] Li, X., Lu, T. & Song, P. Transmission characteristics of the rough surface scattering channel for wireless ultraviolet communication in a cemented ground scenario. *Appl. Opt.* **62**(17), 4591–4599 (2023). 37707156 10.1364/AO.482957

[2] Wang, H., Zhang, Z., Zhu, B., Dang, J., Wu, L., Wang, L. & Zhang, Y. Performance of wireless optical communication with reconfigurable intelligent surfaces and random obstacles. *arXiv preprint *arXiv:2001.05715 (2020).

[3] Qamar, H. H., Hussein, K. F. A., & El-Mashade, M. B. Assessment of signal strength in indoor optical wireless communications using diffuse infrared radiation. in *2019 36th National Radio Science Conference (NRSC)* 108–117 (IEEE, 2019)

[4] Xu, Qi. et al. Joint probabilistic shaping and pre-equalization for hollow-core fiber transmission using end-to-end learning. *Opt. Lett.* **50**(5), 1679–1682 (2025). 40020013 10.1364/OL.544447

[5] Wang, S. et al. SNR enhanced OAM mode division multiplexing with in-band noise modulation based on cascade delta-sigma modulation. *Opt. Express* **33**(1), 1058–1068 (2025). 39876285 10.1364/OE.544348

[6] Zhou, Ji. et al. Low-PAPR layered/enhanced ACO-SCFDM for optical-wireless communications. *IEEE Photonics Technol. Lett.* **30**(2), 165–168 (2017).

[7] Zhong, J., Zhou, Ji., Liu, W. & Qin, J. Orthogonal time-frequency multiplexing with 2D Hermitian symmetry for optical-wireless communications. *IEEE Photonics J.* **12**(2), 1–10 (2020).

[8] Zhang, Li. et al. High-speed multi-user optical wireless communication between VCSEL-integrated electronic devices. *Opt. Commun.* **486**, 126774 (2021).

[9] Harvey, J. E., Goshy, J. J. & Pfisterer, R. N. Modeling stray light from rough surfaces and subsurface scatter. *Reflect., Scatter., Diff. Surf. IV, Int. Soc. Opt. Photonics* **9205**, 92050I (2014).

[10] Beckmann, P. Scattering by composite rough surfaces. *Proc. IEEE* **53**(8), 1012–1015 (1965).

[11] Hyde, M. W., Basu, S., Spencer, M. F., Cusumano, S. J. & Fiorino, S. T. Physical optics solution for the scattering of a partially-coherent wave from a statistically rough material surface. *Opt. Express* **21**(6), 6807–6825 (2013). 23546063 10.1364/OE.21.006807

[12] Tian, J., Tong, J., Shi, J. & Gui, L. A new approximate fast method of computing the scattering from multilayer rough surfaces based on the Kirchhoff approximation. *Radio Sci.* **52**(2), 186–193 (2017).

[13] Thorsos, E. I. The validity of the Kirchhoff approximation for rough surface scattering using a Gaussian roughness spectrum. *J. Acoust. Soc. Am.* **83**(1), 78–92 (1988).

[14] Voti, R. et al. Light scattering from a rough metal surface: theory and experiment. *J. Opt. Soc. Am. B* **26**(8), 1585–1593 (2009).

[15] Sanchez-Gil, J. A. & Nieto-Vesperinas, M. Light scattering from random rough dielectric surfaces. *J. Opt. Soc. Am. A* **8**(8), 1270–1286 (1991).

[16] Nordam, T., Letnes, P. & Simonsen, I. Numerical simulations of scattering of light from two-dimensional rough surfaces using the reduced Rayleigh equation. *Front. Phys.* **1**, 8 (2013).

[17] Elfouhaily, T. M. & Guérin, C. A. A critical survey of approximate scattering wave theories from random rough surfaces. *Waves Random Media* **14**(4), R1–R40 (2004).

[18] Soliman, S. A. M., Farahat, A. E., Hussein, K. F. A. & Ammar, A. A. Spatial domain generation of random surface using Savitzky-Golay filter for simulation of electromagnetic polarimetric systems. *Appl. Comput. Electromagn. Soc. J.* **34**(1), 148–161 (2019).

[19] Tang, K., Dimenna, R. & Buckius, R. Regions of validity of the geometric optics approximation for angular scattering from very rough surfaces. *Int. J. Heat Mass Transf.* **40**(1), 49–59 (1996).

[20] Tang, K. & Buckius, R. The geometric optics approximation for reflection from two-dimensional random rough surfaces. *Int. J. Heat Mass Transf.* **41**(13), 2037–2047 (1998).

[21] Ishimaru, A. & Chen, J. Scattering from very rough surfaces based on the modified second-order Kirchhoff approximation with angular and propagation shadowing. *J. Acoust. Soc. Am.* **88**(4), 1877–1883 (1990).

[22] Hussein, K. F. A. Fast computational algorithm for EFIE applied to arbitrarily-shaped conducting surfaces. *Progr. Electromagn. Res.* **68**, 339–357 (2007).

[23] Shi, F., Choi, W., Lowe, M. J. S., Skelton, E. A. & Craster, R. V. The validity of Kirchhoff theory for scattering of elastic waves from rough surfaces. *Proc. R. Soc. A: Math. Phys. Eng. Sci.* **471**(2178), 20140977 (2015).

